# Inhibition of the ubiquitin-proteasome system by an NQO1-activatable compound

**DOI:** 10.1038/s41419-021-04191-9

**Published:** 2021-10-06

**Authors:** Tatiana A. Giovannucci, Florian A. Salomons, Martin Haraldsson, Lotta H. M. Elfman, Malin Wickström, Patrick Young, Thomas Lundbäck, Jürgen Eirich, Mikael Altun, Rozbeh Jafari, Anna-Lena Gustavsson, John Inge Johnsen, Nico P. Dantuma

**Affiliations:** 1 Department of Cell and Molecular Biology (CMB), Karolinska Institutet, Stockholm, Sweden; 2 Chemical Biology Consortium Sweden (CBCS), Science for Life Laboratory, Division of Translational Medicine and Chemical Biology, Department of Medical Biochemistry and Biophysics, Karolinska Institutet, Solna, Stockholm, Sweden; 3 Childhood Cancer Research Unit, Department of Women’s and Children’s Health, Karolinska Institutet, Stockholm, Sweden; 4 Science for Life Laboratory, Department of Oncology-Pathology, Clinical Proteomics Mass Spectrometry, Karolinska Institutet, Solna, Stockholm, Sweden; 5 Science for Life Laboratory, Department of Medical Biochemistry and Biophysics (MBB), Karolinska Institutet, Solna, Stockholm, Sweden; 6 Science for Life Laboratory, Department of Laboratory Medicine, Karolinska Institutet, Solna, Stockholm, Sweden; 7 Present Address: Mechanistic & Structural Biology, Discovery Sciences, R&D, AstraZeneca, Gothenburg, Sweden; 8 Present Address: Institute of Plant Biology and Biotechnology, University of Muenster, 48143 Muenster, Germany

**Keywords:** Ubiquitylation, Screening

## Abstract

Malignant cells display an increased sensitivity towards drugs that reduce the function of the ubiquitin-proteasome system (UPS), which is the primary proteolytic system for destruction of aberrant proteins. Here, we report on the discovery of the bioactivatable compound CBK77, which causes an irreversible collapse of the UPS, accompanied by a general accumulation of ubiquitylated proteins and caspase-dependent cell death. CBK77 caused accumulation of ubiquitin-dependent, but not ubiquitin-independent, reporter substrates of the UPS, suggesting a selective effect on ubiquitin-dependent proteolysis. In a genome-wide CRISPR interference screen, we identified the redox enzyme NAD(P)H:quinone oxidoreductase 1 (NQO1) as a critical mediator of CBK77 activity, and further demonstrated its role as the compound bioactivator. Through affinity-based proteomics, we found that CBK77 covalently interacts with ubiquitin. In vitro experiments showed that CBK77-treated ubiquitin conjugates were less susceptible to disassembly by deubiquitylating enzymes. In vivo efficacy of CBK77 was validated by reduced growth of NQO1-proficient human adenocarcinoma cells in nude mice treated with CBK77. This first-in-class NQO1-activatable UPS inhibitor suggests that it may be possible to exploit the intracellular environment in malignant cells for leveraging the impact of compounds that impair the UPS.

## Introduction

Proteotoxic stress is a condition that is observed in many cancer types and can be capitalized in anticancer regimens [1]. The underlying causes of proteotoxic stress in malignant cells are multifactorial and inherently linked to their hyperactive state, such as an overall increase in protein synthesis and accumulation of aberrant and orphan proteins due to the loss of genome integrity. The ubiquitin-proteasome system (UPS) is the primary proteolytic system in charge of the timely and efficient degradation of aberrant and excessive proteins that are at risk of polluting the intracellular milieu [2]. In malignant cells, the UPS is close to the limits of its maximal capacity and hence its operational efficiency is of critical importance for their survival [3]. The increased demand for clearance of misfolded proteins generates a therapeutic window at which reduction of UPS activity is lethal for cancer cells without severely affecting protein homeostasis in healthy cells [4]. To date, three proteasome inhibitors have been clinically approved for treatment of multiple myeloma and mantle cell lymphoma [5]. Despite the overall success of proteasome inhibitors in the treatment of hematological malignancies, several remaining challenges, such as adverse side effects, drug resistance, and poor activity towards solid tumors, motivate the exploration of alternative approaches for inhibiting UPS activity.

Proper functioning of the UPS involves hundreds of proteins, many of which display apparent druggable enzymatic activities [2]. Recent efforts have focused on the development of compounds that inhibit deubiquitylating (DUB) enzymes [6], the ubiquitin-selective unfoldase p97/valosin containing protein (VCP) [7], enzymes involved in ubiquitylation [8], or ubiquitin itself [9, 10]. The integrated nature of the UPS makes it hard to predict alternative strategies for obstructing this complex proteolytic pathway in cancer cells. Therefore, we opted for an unbiased, high-content phenotypic screen in a pursuit for small molecules that cause a general inhibition of the UPS by novel molecular mechanisms. This screen led to the discovery of the bioactivatable UPS inhibitor CBK77 that requires the redox enzyme NAD(P)H:quinone oxidoreductase 1 (NQO1), which is often upregulated in malignant cells [11]. We show that this first-in-class compound modifies ubiquitin conjugates, causes an irreversible global inhibition of ubiquitin-dependent proteasomal degradation, and induces caspase-dependent cell death.

## Materials and methods

### Compound screen

The compound library consisted of a diverse set of 5720 small molecules from the Chemical Biology Consortium Sweden (www.cbcs.se) The set can be described as a chemically diverse representation of a larger collection of lead- and drug-like compounds, designed to afford straightforward screen follow-up through testing of close analogs to hit compounds. Details of the primary compound screen can be found in Supplementary Table S1.

### **Reagents**

*Chemicals*: Epoxomicin (Enzo Life Sciences, BML-PI127). Bortezomib (Sigma-Aldrich, 5043140001). Q-VD-OPh (OPH001, R&D systems). Necrostatin-1 (Enzo Life Sciences, BML-AP309). Dicoumarol (Sigma-Aldrich, 287897), ES936 (Santa Cruz Biotechnologies, sc-36273). *Antibodies*: β-actin 1:5000 (Abcam, ab8226), GAPDH 1:5000 (Abcam, ab9485), GFP 1:5000 (Abcam, ab290), p53 1:1000 (DO-1, Santa Cruz Biotechnologies, sc-126), Ubiquitin 1:1000 (Cell Signaling Technologies, #3933), NQO1 1:1000 (A180, Novus Biologicals, NB200-209), FLAG 1:2000 (M2, Sigma-Aldrich, F3165), CRISPR-Cas9 antibody (7A9-3A3, Abcam, ab202580). Secondary HRP-conjugated antibodies were sheep anti-mouse (NA931) and goat anti-rabbit (NA934) from GE Healthcare, or LI-COR 680CW goat anti-rabbit (926-68071) and 800CW goat anti-mouse (926-32210) and used at 1:5000 dilution.

### **Plasmids**

The pcDNA3 NQO1-FLAG (Addgene #61729) and pEIFIRES NQO1 (Addgene #61735) plasmids were a gift from Yosef Shaul. For bacterial expression of recombinant GST-NQO1, the pGEX-6P-1 vector was linearized using the BamHI/XhoI restriction sites. NQO1 was amplified from the pEIFIRES vector by PCR, in which the following primers were used to introduce overhangs that were complementary to the linearized pGEX-6P-1: 5’ TCT GTT CCA GGG GCC CCT GGG ATC CAT GGT CGG CAG AAG AGC AC-3’ and 5’- GTC AGT CAC GAT GCG GCC GCT CGA GTC ATT TTC TAG CTT TGA TCT GGT TG-3’. Gibson assembly was performed following the manufacturer’s instructions (NEBuilder High-Fidelity DNA Assembly Cloning Kit, Nordic Biolabs).

### Cell culture

The human melanoma MelJuSo cells, human cervical cancer HeLa, and the human breast carcinoma MDA-MB-231 cells were maintained in DMEM. LS174T cells were maintained in RPMI 1640 medium. All media contained high glucose, GlutaMAX and sodium pyruvate, and were supplemented with 10% fetal bovine serum. All cells were maintained at 37 °C in a humidified 5% CO_2_ atmosphere and tested periodically for *Mycoplasma* infection by PCR. MelJuSo cells stably expressing Ub-YFP, Ub-R-GFP, YFP-CL1, and CD3δ-YFP were previously generated [12]. The ZsGreen-ODC (ornithine decarboxylase) cells were previously generated by transfection of MelJuSo cells with the ZsProsensor-1 plasmid (BD Bioscience Clontech) [13]. MDA-MB-231 cells were a kind gift from Galina Selivanova (Karolinska Institutet, Sweden) and were used to generate stably expressing NQO1^FLAG^ by transfection with the NQO1-FLAG plasmid. After 16 h, selection was started (1 mg/ml geneticin). Clones expressing NQO1^FLAG^ were isolated and validated by western blotting. For the CRISPR/Cas9 screen, the MelJuSo cell line was made to stably express the Cas9 nuclease. In brief, a construct coding for Cas9 and blasticidin under the control of the EF1α promoter was introduced by lentiviral transduction. After 2 weeks of blasticidin selection, Cas9 expression was confirmed by western blot. Plasmid DNA and siRNA transfections were performed using lipofectamine 2000 (Thermo Fisher Scientific) according to the manufacturer’s instructions. A list of all the siRNAs used in this study can be found in Supplementary Table S5.

### Cell proliferation assay

Cells were seeded into 96-well plates at 1500–5000 cells/well. Sixteen hours after seeding (ca 60% confluency), cells were treated with the indicated compound concentrations in a serial dilution. DMSO at 1% was used as control. After 48 or 72 h of incubation, WST-1 tetrazolium salt (Sigma–Aldrich,11644807001) was added and incubated for 1 h at 37 °C. The formation of formazan was assessed by measuring the absorbance at 480 nm with the plate reader FLUOStar OPTIMA (BMG Labtech; Ortenberg, Germany).

### Western blotting

Equal amounts of cells were lysed in 1× SDS sample buffer (Tris-HCl 0.3 M pH 6.8, 2% SDS, 17.5% glycerol, bromophenol blue) containing 10% NuPAGE reducing agent (Thermo Fisher Scientific, NP0004) and lysates were boiled at 95 °C for 5 min. Cell protein extracts were resolved by Bis-Tris polyacrylamide gel electrophoresis gels (Thermo Fisher Scientific, 4–12% gradient gels [NP0323]) and run in either MOPS (Thermo Fisher Scientific, NP0001) or MES buffer (Thermo Fisher Scientific, NP0002). Proteins were transferred onto PVDF 0.45 µm or nitrocellulose membranes (GE Healthcare, 10600023) in a Tris-glycine transfer buffer (25 mM Tris, 192 mM glycine) containing 20% methanol. After blocking in Tris-buffered saline (TBS) (Statens Veterinärmedicinska Anstalt 303252) containing 5% non-fat milk and 0.1% Tween-20 (Sigma–Aldrich, P9416), membranes were incubated with primary antibodies, washed with TBS-0.1% Tween-20 and incubated with secondary HRP-linked antibodies (GE Healthcare, NA934V and NA931V). Detection was performed by enhanced chemiluminiscence (Amersham ECL reagents, GE Healthcare, RPN2106) on X-ray films (Fujifilm). Alternatively, secondary antibodies coupled to near-infrared fluorescent dyes (LI-COR, 926-68070 and 926-68071) were used, and membranes scanned with an Odyssey scanner (LI-COR, Lincoln, NE, USA) and analyzed with Image Studio Lite analysis software version 5.2 (LI-COR).

### CRISPR/Cas9 interference screen

#### Brunello-UMI Library

The genome-wide Brunello sgRNA library [14] was resynthesized to include Unique Molecular Identifiers [15]. Guides were cloned in pool and packaged into lentivirus. Lentiviral backbone was based on lentiGuide-Puro (Addgene # 52963), with AU-flip as in [16].

#### Screen

Cas9-expressing cells were transduced with the Brunello-UMI library at an approximate MOI of 0.4 and 1000 cells/guide in 2 µg/ml polybrene. Transduced cells were selected with puromycin (2 µg/ml) from post-transduction day 2–7 and then split into two replicates cultured independently until 16 days after transduction. Cells were treated with either DMSO or CBK77 at 25 µM. Two replicates of CBK77-treated cells were performed in parallel with a common DMSO-treated sample (the number of CBK77-treated cells and DMSO-treated cells corresponded to ~200-fold and 500-fold library coverage, respectively). When less than 10% of the CBK77-treated cells were left, cells were harvested and genomic DNA was isolated using the QIAamp DNA Blood Maxi kit (Qiagen), and guide and UMI sequences were amplified by PCR. Sequencing was performed on an Illumina HiSeq instrument. Next Generation Sequencing (NGS) data were analyzed with MaGeCK [17] and by UMI lineage dropout analysis [15]. We considered interesting genes those which guides had an average log-fold change (LFC) higher than 1 and had a false discovery rate (FDR) lower than 0.1. A summary of the results can be found in Supplementary Table S3.

### Protein purification

BL21 *E. coli* bacteria were transformed with the pGEX-6P-1 plasmid encoding GST-NQO1. Overnight cultures of individual colonies were grown at 37 °C in the presence of 100 μg/ml ampicillin. Bacteria were grown at 37 °C in 500 ml LB until reaching OD600 = 0.6, when protein expression was induced by 1 mM IPTG for 3.5 h at 30 °C. Bacteria were harvested and resuspended in GST lysis buffer (50 mM HEPES pH 7.5, 500 mM NaCl, 10% glycerol, 1 mM MgCl_2_, 10 mM DTT) and lysed by sonication. The lysate was cleared and incubated with equilibrated glutathione sepharose 4B beads (GE Healthcare, 17075605) overnight at 4 °C. Beads were washed in GST lysis buffer and GST-NQO1 was eluted for 4 h at 4 °C in elution buffer (50 mM Tris pH 7.5, 10 mM reduced glutathione, 10 mM DTT). To obtain untagged NQO1, beads were washed in PreScission cleavage buffer (50 mM Tris pH 7.5, 150 mM NaCl, 1 mM EDTA, 1 mM DTT) and protein was eluted by ca 40 units PreScission protease (GE Healthcare, 27084301) for 4 h at 4 °C under rotation. The purity of the resulting protein was analyzed on a Coomassie gel.

### NQO1 in vitro assay

NQO1 activity was assessed by measuring the levels of NAD(P)H via absorbance at 340 nm in a Versamax plate reader (Molecular Devices; San Jose, CA, USA) at 37 °C. The reaction mixture in a final volume of 200 µl contained 50 mM ammonium bicarbonate, 0.1 mg/ml BSA, 2 mM EDTA, 200 µM NAD(P)H as electron donor (Sigma–Aldrich, N7505) and 2 µg of recombinant NQO1 (for final enzyme concentration of 0.5 µM). Positive and negative controls were included by adding 400 µM of the NQO1 known substrate menadione (Sigma–Aldrich, M5625) in the presence or absence of the NQO1 inhibitor dicoumarol at 100 µM, respectively. To assess whether the compounds were substrates of NQO1, 100 µM of the indicated compounds were added instead of menadione in the absence or presence of dicoumarol to measure specific NQO1 activity. Measurements were made every 10 sec for 20 min. For analytical high-performance liquid chromatography with diode array detection (DAD) and electrospray ionization mass spectrometry (HPLC-MS), the in vitro reactions were filtered using Amicon Ultra-0.5 centrifugal filter devices with a 30 kD molecular weight cutoff (Merck-Millipore) applying centrifugation for 10 min at 13,000 × *g*. 100 µl of flow-through samples were injected on an Agilent/HP 1200 system 6110 mass spectrometer with electrospray ionization (ESI+). Absorbance was monitored at 305 ± 90.

### Proteasomal activity

After treatments, cells were trypsinized and lysed (25 mM HEPES pH 7.2, 50 mM NaCl, 1 mM MgCl_2_, 1 mM ATP, 1 mM DTT, 10% glycerol, 1% Triton X-100) for 30 min at 4 °C. Ten micrograms protein in 100 μl reaction buffer were mixed with 100 μM Suc-Leu-Leu-Val-Tyr-AMC (Affiniti, P802). In one well, 500 nM epoxomicin was added additionally. Samples were analyzed in a microplate reader (FLUOStar OPTIMA, BMG Labtech; Ortenberg, Germany) at 380 nm/440 nm every minute for 1 h.

### Cellular DUB activity

After treatments, cells were trypsinized and lysed (25 mM HEPES pH 7.2, 50 mM NaCl, 1 mM MgCl_2_, 1 mM ATP, 1 mM DTT, 10% glycerol, 1% Triton X-100). Protein concentrations were measured with the Bradford assay (Bio-Rad). Ten micrograms protein were mixed with 80 μl reaction buffer (lysis buffer without Triton X-100) and 10 μl ubiquitin-AMC (Enzo Life Sciences, BML-SE211-0025) for a final concentration of 1 μM. Samples were analyzed in a microplate reader (FLUOStar OPTIMA, BMG Labtech; Ortenberg, Germany) at 355 nm/460 nm every 30 sec for 1 h.

### DUB profiling

Labelling of DUBs with activity-based probe was carried out as previously described [18]. In brief, after treatments, cells were lysed (50 mM Tris buffer pH 7.4, 150 mM NaCl, 5 mM EDTA, 0.5% NP-40). Lysates were incubated on ice for 45 min and cleared by centrifugation (10,000 × *g* for 20 min at 4 °C). Protein concentrations were measured by BCA. 100 μg of protein were incubated with 4 µg of HA-tagged ubiquitin glycine vinyl methylester (HA-UbVME) or HA-tagged ubiquitin bromoethylamine (HA-UbBr2) for 2 h at 37 °C. A DMSO-treated lysate without probe was included as a background control. After incubating, samples were denatured by the addition of LDS-sample buffer and boiled at 95 °C for 5 min. Samples were analyzed by SDS-PAGE and immunoblotting with anti-HA and specific DUB antibodies.

### *CLICK* chemistry and analytical labelling

Cells were washed with phosphate-buffered saline (PBS) and the pellets lysed in lysis buffer (1% NP-40, 100 mM NaCl, 50 mM HEPES pH 7.2). Following centrifugation (10 min, 16,000 × *g*, 4 °C), supernatants were supplemented with 50 µM TAMRA-azide (Jena Bioscience, CLK-FA008-1), 5 mM sodium ascorbate (Sigma–Aldrich, A7631), 5 mM aminoguanidine-HCl (Sigma–Aldrich, 396494), 0.1 mM CuSO4 (Sigma–Aldrich, PHR1477), and 0.5 mM THPTA (Jena Bioscience, CLK-1010-25), with gentle vortexing. The reaction mixtures were incubated at RT for 30 min, light protected. For gel electrophoresis, 4× LDS-sample buffer was added. Samples were resolved by SDS-PAGE. In-gel fluorescence was recorded using a ChemiDoc MP Imaging System (Bio-Rad Laboratories; Hercules, CA, USA) equipped with 695/55 (Cy5, for visualization of the Precision Plus Protein Standard, Bio-Rad) and 605/50 (Cy3/TAMRA) filters and coupled to Image Lab v 5.0 analysis software (Bio-Rad).

### Streptavidin-beads pulldown using the TFL TAMRA-azide-biotin

Cells previously seeded into 10 cm dishes were treated with either CBK77^CLICK^ at 20 µM or with DMSO 0.2% for 1.5 h. Cells were then lysed in the aforementioned lysis buffer. Following centrifugation (16,000 × *g*, 15 min, 4 °C), supernatants were supplemented with 50 µM trifunctional linker TAMRA-azide-PEG3-biotin (TFL, Jena Bioscience, CLK-1048-5), and *click* chemistry performed as described. Proteins were precipitated by the addition of a five-fold volume excess of acetone and incubated overnight at −20 °C. Following centrifugation (16,000 × *g*, 30 min, 4 °C), the supernatant was discarded, and pellets washed two times with prechilled methanol. Subsequently, pellets were dissolved in 0.2% SDS in 25 mM sodium bicarbonate by sonication and incubated under gentle mixing with 100 μL of Dynabeads M-280 streptavidin (Thermo Fisher Scientific, 11206D) for 2 h at 4 °C. The beads were washed three times with 25 mM ammonium bicarbonate/0.2% SDS, twice with 6 M urea, and three times with 25 mM ammonium bicarbonate. For gel analysis, 15 μL 2× SDS loading buffer were added and the proteins released for SDS-PAGE by 10 min incubation at 95 °C.

### Proteomics

The bead-bound proteins after streptavidin-beads pulldown were reduced in 1 mM DTT at 37 °C for 1 h. Next, samples were alkylated by incubation in the dark at RT in 5 mM chloroacetamide for 10 min. After, any remaining chloroacetamide was quenched by the addition of 5 mM DTT. Digestion was carried out by the addition of 0.4 µg Trypsin (sequencing grade modified, Pierce) overnight at 37 °C. Peptides were collected and dried using a speedvac. Peptides were then dissolved in 30 µl 3% acetonitrile + 0.1% formic acid and transferred to an MS-vial.

#### LC-ESI-MS/MS

LC-MS analysis was performed using a Dionex UltiMate 3000 RSLCnano System coupled to a Q-Exactive mass spectrometer (Thermo Scientific). Five microliters per sample were injected. Samples were trapped on a C_18_ guard desalting column (Acclaim PepMap 100, 75 µm × 2 cm, nanoViper, C_18_, 5 µm, 100 Å), and separated on a 50 cm long C_18_ column (Easy spray PepMap RSLC, C_18_, 2 µm, 100 Å, 75 µm × 15 cm). The nano capillary solvent A was 95% water, 5% DMSO, 0.1% formic acid; and solvent B was 5% water, 5% DMSO, 95% acetonitrile, 0.1% formic acid. At a constant flow of 0.25 μl min^−1^, the curved gradient went from 6% B up to 43% B in 180 min, followed by a steep increase to 100% B in 5 min.

FTMS master scans with 60,000 resolution (and mass range 300–1500 *m/z*) were followed by data-dependent MS/MS (30,000 resolution) on the top 5 ions using higher energy collision dissociation (HCD) at 30% normalized collision energy. Precursors were isolated with a 2 *m/z* window. Automatic gain control (AGC) targets were 1e6 for MS1 and 1e5 for MS2. Maximum injection times were 100 ms for MS1 and MS2. The entire duty cycle lasted ~2.5 s. Dynamic exclusion was used with 60 s duration. Precursors with unassigned charge state or charge state 1 were excluded. An underfill ratio of 1% was used.

#### Peptide and protein identification

The MS raw files were searched using Sequest HT and validated with Percolator with Proteome Discoverer 1.4 (Thermo Scientific) against human UniProt database (downloaded 2015-04-06) and filtered to a 1% FDR cutoff. We used a precursor ion mass tolerance of 15 ppm, and product ion mass tolerances of 0.02 Da for HCD-FTMS. The algorithm considered tryptic peptides with maximum two missed cleavages; carbamidomethylation (C) as fixed modifications; oxidation (M) as variable modifications.

### Gene ontology

Enriched molecular functions in the proteomics dataset were calculated using String (https://string-db.org/). The minimum required interaction score was selected to high confidence (0.7) and only ‘Experiment’ and ‘Databases’ were considered. Results are displayed in Supplementary Table S4.

### Microscopy

Widefield fluorescent images were taken with the ImageXpress Micro XLS microscope (Molecular Devices; San Jose, CA, USA) equipped with ×10 or ×20 air objectives (NA 0.45 Nikon ELWD) and a dichroic mirror set for DAPI, FITC, Cy3, Cy5, and Texas Red. Live-cell imaging experiments for 24 h were performed in Leibovitz’s L-15 medium (Life Technologies, 11415-064) at 37 °C. Imaging was performed using the MetaXpress software (Molecular Devices). For confocal images, the Zeiss LSM880 microscope (Zeiss; Oberkochen, Germany) equipped with a ×63 Plan-A (1.4 NA) oil-immersion lens was used. Images were recorded using the ZEN 2012 software (Zeiss). Images were analyzed with either in-built pipelines in the MetaXpress software (Molecular Devices), custom-made pipelines in CellProfiler (www.cellprofiler.org*)* or ImageJ (https://imagej.nih.gov/ij/). ImageJ was also used for the processing of representative images.

### In vivo experiments

Nineteen NMRI nu/nu female mice 6–8 weeks of age (Taconic) were anesthetized and 10 million LS174T cells were subcutaneously injected into a flank of the mouse. When the tumors reached a minimum volume of 0.1 mL, mice were randomly assigned to either vehicle (*n* = 10) or compound (*n* = 9) treatment (day 1). The mice were treated daily for a maximum of 16 days with intraperitoneal injection of either vehicle (solvent only) or compound at 10 mg/kg concentration. At day 16, animals were sacrificed, and tumors weighted and cut into two parts, which were fixed in 4% formalin (for standard FFPE processing) or were frozen at −80 °C. Tumor size was assessed every two days using a caliper and tumor volumes were calculated using the formula length × width^2^ × 0.44. For calculating the tumor growth slopes, tumor volumes were turned into logarithmic scale and fitted into a simple linear regression model [19]. Animals were maintained at a maximum of six per cage and given sterile water and food ad libitum.

### Statistical analysis

Statistical analyses were performed using RStudio, Python or GraphPad Prism version 8.3. To test for Gaussian distribution, the D’Agostino & Pearson normality test was used. If the normality test was passed, data were analyzed by Student’s unpaired *t*-test (two groups) or by ANOVA (more than two groups). If the data were not normally distributed, statistical analysis was performed using the nonparametric Mann–Whitney test (two groups) or Kruskal–Wallis test for multiple comparisons, with Dunnett’s or Tukey’s test to adjust for multiple comparisons. Grubbs’ test was used for the detection of outliers. Adjusted *p*-values are shown. For the in vivo experiment, no blinding was performed. No sample size calculation was performed. Sample size was based on previous experiments with the LS174T cell line on testing drug effects. Data are shown as mean from at least 2–3 independent experiments. Error bars represent ±SD (standard deviation) or SEM (standard error of the mean), as indicated in each figure legend. The following *p*-values were considered significant: *P ≤ 0.05; **P ≤ 0.01; ***P ≤ 0.001; ****P ≤ 0.0001.

### Synthetic procedures for chemical compounds

#### General methods for chemistry

All evaporations were carried out in vacuo with a rotary evaporator at 10–30 mmHg. Analytical samples were dried under high vacuum (1–5 mmHg) at RT. Thin layer chromatography (TLC) was performed on silica gel plates (Merck) with fluorescent indicator. Spots were visualized by UV light (214 and 254 nm). Flash chromatography was performed using silica gel 60 (Merck). 1H NMR spectra were recorded using a Bruker DPX400 spectrometer (400 MHz) using deuterated solvents. All compounds evaluated in biological tests were purified to ≥95% as determined by HPLC-MS on an Agilent/HP 1200 system 6110 mass spectrometer with electrospray ionization (ESI+). Absorbance was monitored at 305 ± 90 and 254 nm.

#### Analytical HPLC-MS

System A: Column ACE 3 C8, 3 µm, 50 × 3.0 mm maintained at 40 °C. 0.1% (v/v) trifluoroacetic (TFA) in H_2_O and acetonitrile were used as mobile phases at a flow rate of 1 mL/min, with a gradient time of 3 min.

System B: Column Xterra MSC18, 3.5 µm, 50 × 3.0 mm maintained at 40 °C. H_2_O (containing 10 mM ammonium bicarbonate (NH_4_HCO_3_); pH = 10) and acetonitrile were used as mobile phases at a flow rate of 1 mL/min, with a gradient time of 3 min.

#### Preparative HPLC was performed on a Gilson HPLC system

Column ACE C8, 5 µm (150 × 30 mm); 0.1% TFA (v/v) in H_2_O and acetonitrile were used as mobile phases at a flow rate of 35 mL/min with a gradient time of 7 min.

The nomenclature of structures was determined using the “Structure to Name” function of MarvinSketch Carbon v.5 and choosing “preferred IUPAC name”.

##### N‐(6‐ethoxy‐1,3‐benzothiazol‐2‐yl)‐5‐nitrofuran‐2‐carboxamide (CBK006377, “CBK77” in the main text)



1-Propanephosphonic acid cyclic anhydride (2.2 mL, 3.7 mmol) was added to a solution of 5-nitro-2-furanoic acid (124 mg, 0.64 mmol) in dimethylformamide (DMF). After 10 min, 2.2 mL trimethylamine (Et_3_N) (15.9 mmol) and 6-ethoxy-1,3-benzothiazol-2-amine (100 mg, 0.64 mmol) and the mixture left stirring for 1 h were after ethyla acetate (EtOAc) and H_2_O were added. The organic phase was washed with sodium carbonate (Na_2_CO_3_) (aq, sat.) whereby a solid formed that was filtered off and dissolved in H_2_O and methanol (MeOH). Hydrochloric acid (HCl) (1 M) was added dropwise and the final product precipitated as a beige solid. Yield 60 mg. ^1^H NMR (400 MHz, DMSO-d_6_) d = 13.51–13.08 (m, 1 H), 7.82 (br. s., 2 H), 7.60 (br. s., 2 H), 7.13–6.99 (m, 1 H), 4.08 (q, *J* = 6.8 Hz, 4 H), 1.36 (t, *J* = 6.8 Hz, 3 H). ^13^C NMR (101 MHz, DMSO-*d*_6_) δ ppm 14.61 (s, 1 C) 63.64 (s, 1 C) 105.73 (s, 1 C) 113.31 (s, 1 C) 115.71 (s, 1 C) 117.81 (s, 1 C) 129.57 (s, 1 C) 133.41 (s, 1 C) 152.19 (s, 1 C) 155.69 (s, 1 C). LC-MS: *m/z* calculated for C14H12N3O5S: [M + H]^+^, 334.05; found, 334.1

##### N‐(4‐methoxy‐1,3‐benzothiazol‐2‐yl)‐5‐nitrofuran‐2‐carboxamide (CBK085907, “CBK07” in the main text)



To a solution of 5-nitrofuran-2-carboxylic acid (62 mg, 0.40 mmol) in dry DMF (2 mL) were added successively N-methylmorpholine (96 µL, 0.88 mmol) and isobutyl chloroformate (58 µL, 0.44 mmol). The solution was stirred for 15 min and then 4-methoxy-1,3-benzothiazol-2-amine (72 mg, 0.40 mmol) was added and the reaction was left at RT stirring for 2 h. H_2_O (2 mL) was added and the product was filtered off and washed with hot water to of a yellow solid. Yield 65 mg. ^1^H NMR (400 MHz, DMSO-d6) δ ppm 3.93 (s, 3 H) 7.04 (d, *J* = 8.06 Hz, 1 H) 7.30 (t, *J* = 7.98 Hz, 1 H) 7.57 (d, *J* = 7.90 Hz, 1 H) 7.82 (d, *J* = 3.95 Hz, 1 H) 7.88 (br. s., 1 H) 13.57 (br. s., 1 H) ^13^C NMR (101 MHz, DMSO-d6) δ ppm 55.78 (s, 1 C) 107.76 (s, 1 C) 113.35 (s, 1 C) 113.63 (s, 1 C) 117.80 (s, 1 C) 124.83 (s, 1 C) 132.62 (br. s, 1 C) 146.94 (br. s, 1 C) 151.44 (br. s, 1 C) 152.34 (s, 1 C).) LC-MS: *m/z* calculated for C13H10N2O5S: [M + H]^+^, 320.03; found, 320.0

##### Prop‐2‐yn‐1‐yl N‐[2‐(5‐nitrofuran‐2‐amido)‐1,3‐benzothiazol‐6‐yl]carbamate (CBK293384, “CBK77^CLICK^” in the main text)



Propargyl chloroformate (30 µL, 0.31 µmol) was added at RT to a solution of N-(6-aminobenzo[d]thiazol-2-yl)-5-nitrofuran-2-carboxamide hydrochloride (60 mg, 0.18 mmol) and methylmorpholine (90 µL, 0.65 mmol) in DMF (1 mL) and the mixture was stirred overnight. Aqueous MeOH was added and the product was filtered off. Washing with aqueous MeOH and drying in vacuum gave the title compound as an orange solid. Yield 38 mg. ^1^H NMR (400 MHz, DMSO-*d*_6_) δ ppm 3.58 (t, *J* = 2.45 Hz, 1 H) 4.78 (d, *J* = 2.37 Hz, 2 H) 7.48 (dd, *J* = 8.77, 2.13 Hz, 1 H) 7.65 (d, *J* = 8.69 Hz, 1 H) 7.77 (d, *J* = 3.47 Hz, 1 H) 7.81 (dd, *J* = 3.95, 1.00 Hz, 1 H) 8.09–8.12 (m, 1 H) 10.05 (s, 1 H) 13.42 (br. s., 1 H). ^13^C NMR (101 MHz, DMSO-*d*_6_) δ ppm 52.06 (s, 1 C) 77.67 (s, 1 C) 78.92 (s, 1 C) 110.87 (br. s., 1 C) 113.36 (s, 1 C) 117.94 (br. s., 1 C) 118.38 (br. s., 1 C) 135.35 (s, 1 C) 152.31 (s, 1 C) 152.73 (s, 1 C)LC-MS: *m/z* calculated for C16H11N4O6S: [M + H]^+^, 387.04; found, 387.0

#### Synthesized compounds for SAR analysis

##### 5‐nitro‐N‐(1,3‐thiazol‐2‐yl)furan‐2‐carboxamide (CBK260866)



5‐Nitrofuran‐2‐carboxylic acid (70 mg, 0.13 mmol) was treated with thionyl chloride (2 mL) at reflux for 5 h and then the volatile was evaporated, and the residue was dried in vacuum. The residue was dissolved in dichloromethane (21 mL) and 1/3 of this solution (7 mL) was added to 1,3‐thiazol‐2‐amine (10 mg, 0.10 mmol) and then pyridine (100 µL) was added and the mixture was stirred overnight at RT. The resulting solid was filtered off and washed with MeOH to afford the title compound as a yellow solid. Yield 10 mg. ^1^H NMR (400 MHz, DMSO-d6) δ ppm 7.30 (d, *J* = 3.76 Hz, 1 H) 7.59 (d, *J* = 3.76 Hz, 1 H) 7.73 (br. s., 1 H) 7.80 (d, *J* = 3.76 Hz, 1 H) 13.31 (br. s., 1 H) LC-MS: *m/z* calculated for C8H6N3O4S: [M + H]^+^, 240.01; found, 240.1

##### N‐(6‐methoxy‐1,3‐benzothiazol‐2‐yl)furan‐2‐carboxamide (CBK260867)



Diisopropylethylamine (21 µL, 0.12 mmol) was added to a mixture of 2-(1H-Benzotriazole-1-yl)-1,1,3,3-tetramethyluronium hexafluorophosphate (HBTU) (45 mg, 0.12 mmol) and furan‐2‐carboxylic acid (11 mg. 0.10 mmol) in DMF (0.2 mL). After stirring for 15 min at RT, 6‐methoxy‐1,3‐benzothiazol‐2‐amine (19 mg, 0.10 mmol) was added stirring continued over 72 h. The reaction product was purified by preparative HPLC to give the title compound as a white solid. Yield 19 mg. ^1^H NMR (400 MHz, DMSO-*d*_6_) δ ppm 1.36 (t, *J* = 6.95 Hz, 3 H) 4.08 (q, *J* = 6.95 Hz, 2 H) 6.73–6.78 (m, 1 H) 7.04 (dd, *J* = 8.77, 2.61 Hz, 1 H) 7.58 (d, *J* = 2.53 Hz, 1 H) 7.65 (d, *J* = 8.85 Hz, 1 H) 7.70 (d, *J* = 3.48 Hz, 1 H) 8.04 (dd, *J* = 1.74, 0.79 Hz, 1 H) 12.12–13.24 (m, 1 H). LC-MS: *m/z* calculated for C14H13N2O3S: [M + H]^+^, 289.06; found, 289.1

##### N‐(1,3‐benzothiazol‐2‐yl)‐5‐nitrofuran‐2‐carboxamide (CBK260868)



Diisopropylethylamine (21 µL, 0.12 mmol) was added to a mixture of HBTU (45 mg, 0.12 mmol) and 5‐nitrofuran‐2‐carboxylic acid (16 mg. 0.10 mmol) in DMF (0.2 mL). After stirring for 15 min at RT, 1,3‐benzothiazol‐2‐amine (15 mg, 0.10 mmol) was added stirring continued over the weekend. Acetonitrile (0.6 mL) and H_2_O (0.2 mL) was added and the mixture was stirred for 30 min and the product was filtered off and washed with acetonitrile: H_2_O (1:1) to give the title compound as a brown solid. Yield 14 mg. ^1^H NMR (400 MHz, DMSO-*d*_6_) δ ppm 7.32–7.41 (m, 1 H) 7.50 (td, *J* = 7.65, 1.25 Hz, 1 H) 7.64–7.89 (m, 3 H) 8.02 (d, *J* = 7.78 Hz, 1 H) 13.56 (br. s., 1 H). LC-MS: *m/z* calculated for C12H8N3O4S: [M + H]^+^, 290.02; found, 290.1

##### N‐(4‐methyl‐1,3‐benzothiazol‐2‐yl)‐5‐nitrofuran‐2‐carboxamide (CBK260869)



Diisopropylethylamine (21 µL, 0.12 mmol) was added to a mixture of HBTU (45 mg, 0.12 mmol) and 5‐nitrofuran‐2‐carboxylic acid (16 mg. 0.10 mmol) in DMF (0.2 mL). After stirring for 15 min at RT, 4‐methyl‐1,3‐benzothiazol‐2‐amine (16 mg, 0.10 mmol) was added stirring continued for 3 h. Acetonitrile (0.4 mL) and H_2_O (1 mL) was added and the mixture was stirred for 30 min and the product was filtered off and washed with acetonitrile:H_2_O (1:1) to give the title compound as a beige solid. Yield 16 mg. ^1^H NMR (400 MHz, DMSO-*d*_6_) δ ppm 2.61 (s, 3 H) 7.22–7.34 (m, 2 H) 7.75–7.87 (m, 2 H) 7.95 (br. s., 1 H) 13.44 (br. s., 1 H). LC-MS: *m/z* calculated for C13H10N3O4S: [M + H]^+^, 304.03; found, 304.1

##### N‐(6‐methyl‐1,3‐benzothiazol‐2‐yl)‐5‐nitrofuran‐2‐carboxamide (CBK260870)



Diisopropylethylamine (21 µL, 0.12 mmol) was added to a mixture of HBTU (45 mg, 0.12 mmol) and 5‐nitrofuran‐2‐carboxylic acid (16 mg. 0.10 mmol) in DMF (0.2 mL). After stirring for 15 min at RT, 6‐methyl‐1,3‐benzothiazol‐2‐amine (16 mg, 0.10 mmol) was added stirring continued for 3 h. Acetonitrile (0.4 mL) and H_2_O (1 mL) was added and the mixture was stirred for 30 min and the product was filtered off and washed with acetonitrile:H_2_O (1:1) to give the title compound as a beige solid. Yield 12 mg. ^1^H NMR (400 MHz, DMSO-*d*_6_) δ ppm 2.42 (s, 3 H) 7.27–7.33 (m, 1 H) 7.59 (br. s., 1 H) 7.73–7.84 (m, 3 H) 13.14–13.95 (m, 1 H). LC-MS: m/z calculated for C13H10N3O4S: [M + H]^+^, 304.03; found, 304.1

##### N-(6-acetamido-1,3-benzothiazol-2-yl)-5-nitrofuran-2-carboxamide (CBK277750)



Acetic anhydride (10 µL, 0.11 mmol) was added at RT to a solution of N-(6-amino-1,3-benzothiazol-2-yl)-5-nitrofuran-2-carboxamide hydrochloride (10 mg, 0.03 mmol) in pyridine (0.2 mL) and dichloromethane (0.5 mL). After 3 h the solvents were evaporated, and the residue was dried in vacuum over the weekend. The residue was washed with H_2_O and a small amount of aqueous MeOH to give the title compound as a red solid. Yield 3 mg. ^1^H NMR (400 MHz, DMSO-*d*_6_) δ ppm 2.07 (s, 3 H) 7.52–7.60 (m, 1 H) 7.65 (br. s., 1 H) 7.74–7.95 (m, 2 H) 8.31 (s, 1 H) 10.15 (s, 1 H) 13.49 (br. s, 1 H). LC-MS: *m/z* calculated for C14H11N4O5S: [M + H]^+^, 347.03¸ 347.1

##### N‐(6‐chloro‐1,3‐benzothiazol‐2‐yl)‐5‐nitrofuran‐2‐carboxamide (CBK277751)



To a solution of 5-nitrofuran-2-carboxylic acid (31 mg, 0.20 mmol) in dry DMF (1 mL) cooled at 0 °C under N_2_, were added successively N-methylmorpholine (48 µL, 0.44 mmol) and isobutyl chloroformate (29 µL, 0.22 mmol). The solution was stirred for 15 min at 0 °C and then 15 min at RT. 6-chloro-1,3-benzothiazol-2-amine (37 mg, 0.20 mmol) was added and the reaction was left at RT stirring. The product was filtered off. Recrystallization from aqueous MeOH gave the title compound as a yellow solid. Yield 19 mg. ^1^H NMR (400 MHz, DMSO-*d*_6_) δ ppm 7.52 (dd, *J* = 8.53, 2.26 Hz, 1 H) 7.76 (br. s., 1 H) 7.84 (d, *J* = 3.76 Hz, 1 H) 7.85–7.96 (br. s, 1 H) 8.18 (d, *J* = 1.51 Hz, 1 H) 13.53 (br. s., 1 H). LC-MS: *m/z* calculated for C12H7ClN3O4S: [M + H]^+^, 323.98¸ found, 324.0

##### N‐(4‐chloro‐1,3‐benzothiazol‐2‐yl)‐5‐nitrofuran‐2‐carboxamide (CBK277752)



To a solution (0 °C) of 5-nitrofuran-2-carboxylic acid (31 mg, 0.20 mmol) in dry DMF (1 mL), were added successively N-methylmorpholine (48 µL, 0.44 mmol) and isobutyl chloroformate (29 µL, 0.22 mmol). The solution was stirred for 15 min at 0 °C and then 15 min at RT. 4-chloro-1,3-benzothiazol-2-amine hydrobromide (53 mg, 0.20 mmol) was added and the reaction was left at RT stirring. The product was filtered off. Recrystallization from aqueous MeOH gave the title compound as a yellow solid. Yield 21 mg. ^1^H NMR (400 MHz, DMSO-*d*_6_) δ ppm 2.61 (s, 3 H) 7.22–7.34 (m, 2 H) 7.75–7.87 (m, 2 H) 7.95 (br. s., 1 H) 13.44 (br. s., 1 H). LC-MS: *m/z* calculated for C12H7ClN3O4S: [M + H]^+^, 324.00¸ found, 324.0

##### N‐(5‐chloro‐1,3‐benzoxazol‐2‐yl)‐5‐nitrofuran‐2‐carboxamide (CBK277754)



To a solution (0 °C) of 5-nitrofuran-2-carboxylic acid (31 mg, 0.20 mmol) in dry DMF (1 mL), were added successively N-methylmorpholine (48 µL, 0.44 mmol) and isobutyl chloroformate (29 µL, 0.22 mmol). The solution was stirred for 15 min at 0 °C and then 15 min at RT. 5-chloro-1,3-benzothiazol-2-amine (34 mg, 0.20 mmol) was added and the reaction was left at RT stirring. The product was filtered off. Purification by preparative HPLC gave the title compound as a brown solid. Yield 5 mg ^1^H NMR (400 MHz, DMSO-*d*_6_) δ ppm 7.37 (dd, *J* = 8.69, 2.05 Hz, 1 H) 7.63 (d, *J* = 18.17 Hz, 2 H) 7.70 (d, *J* = 8.69 Hz, 1 H) 7.79 (d, *J* = 3.79 Hz, 1 H) 13.03 (br. s, 1 H). ^13^C NMR (101 MHz, DMSO-*d*_6_) δ ppm 109.44 (s, 1 C) 113.93 (s, 1 C) 114.62 (s, 1 C) 116.22 (s, 1 C) 120.62 (s, 1 C) 126.75 (s, 1 C) 145.04 (br. s, 1 C) 145.61 (br. s, 1 C) 150.94 (s, 1 C) 161.51 (s, 1 C) LC-MS: *m/z* calculated for C12H7ClN3O5: [M + H]^+^, 308.00¸ found, 308.0

##### N‐(1H‐1,3‐benzodiazol‐2‐yl)‐5‐nitrofuran‐2‐carboxamide (CBK277755)



To a solution (0 °C) of 5-nitrofuran-2-carboxylic acid (31 mg, 0.20 mmol) in dry DMF (1 mL), were added successively N-methylmorpholine (48 µL, 0.44 mmol) and isobutyl chloroformate (29 µL, 0.22 mmol). The solution was stirred for 15 min at 0 °C and then 15 min at RT. 1H-1,3-benzodiazol-2-amine (27 mg, 0.20 mmol) was added and the reaction was left at RT stirring. The product was filtered off. Purification by preparative HPLC gave the title compound as a yellow solid. Yield 4 mg. ^1^H NMR (400 MHz, DMSO-*d*_6_) δ ppm 7.21–7.24 (m, 2 H) 7.32 (d, *J* = 3.79 Hz, 1 H) 7.41–7.45 (m, 2 H) 7.73 (d, *J* = 3.79 Hz, 1 H) 12.72 (br. s., 2 H)LC-MS: *m/z* calculated for C12H9N4O4: [M + H]^+^, 273.05¸ found, 273.1

##### N-(6-amino-1,3-benzothiazol-2-yl)-5-nitrofuran-2-carboxamide hydrochloride (CBK277756)



Methylmorpholine (0.22 mL, 2 mmol) and isobutyl chloroformate (0.26 mL, 2 mmol) were added to a solution of 5-nitrofuran-2-carboxylic acid (0.31 g, 2 mmol) in DMF (4 mL) at 0 °C. The temperature was allowed to reach ca 10 °C during 1 h and tert-butyl N-(2-amino-1,3-benzothiazol-6-yl)carbamate (0.14 g, 0.52 mmol) was added. After 1 h, more tert-butyl N-(2-amino-1,3-benzothiazol-6-yl)carbamate (0.39 g, 1.47 mmol) was added and the reaction was left stirring on at RT. MeOH (1 mL) and H_2_O (1 mL) was added. The mixture was cooled in ice H_2_O and filtered, the solid was washed with 70% cold MeOH. The crystals were dried in vacuum to yield 0.22 g. The material was treated with concentrated HCl (2 mL) at 100 °C for 10 min. After cooling down to RT, the light-yellow solid was filtered and carefully washed with a small amount of cold H_2_O. The solid was dried in vacuum. The color quickly becomes dark brown. Yield 70 mg. ^1^H NMR (400 MHz, DMSO-*d*_6_) δ ppm 7.45 (dd, *J* = 8.53, 2.21 Hz, 1 H) 7.81 (d, *J* = 8.69 Hz, 1 H) 7.84 (d, *J* = 3.95 Hz, 1 H) 7.85–7.89 (m, 1 H) 8.01 (d, *J* = 2.05 Hz, 1 H) 10.38 (br. s., 1 H). ^13^C NMR (101 MHz, DMSO-*d*_6_) δ ppm 113.29 (s, 1 C) 116.12 (s, 1 C) 118.31 (s, 1 C) 121.46 (s, 1 C) 129.06 (s, 1 C) 152.38 (s, 1 C). LC-MS: *m/z* calculated for C12H9N4O4S: [M + H]^+^, 305.03¸ found, 305.1

##### N-(4,5-dichloro-1,3-benzothiazol-2-yl)-5-nitrofuran-2-carboxamide (CBK277757)



DMF (2.5 µL) was added to a stirred mixture of 5-nitrofuran-2-carboxylic acid (50 mg, 0.32 mmol) and oxalyl chloride (50 µL) in dichloromethane (1 mL) at RT. After 30 min the solvents were evaporated, and the residue dried in vacuum. The solid material was dissolved in dichloromethane (4 mL) and treated with 4,5-dichloro-1,3-benzothiazol-2-amine (70 mg, 0.32 mmol). Triethylamine (50 µL) was added and the mixture was stirred at RT overnight. Water and EtOAc was added and the organic phase were evaporated, and the residue purified by preparative HPLC to give the target compound. Yield 3 mg. ^1^H NMR (400 MHz, DMSO-*d*_6_) δ ppm 7.67 (d, *J* = 3.76 Hz, 1 H) 7.76 (d, *J* = 8.78 Hz, 1 H) 7.83 (d, *J* = 4.02 Hz, 1 H) 7.87 (d, *J* = 8.53 Hz, 1 H) 10.84 (s, 1 H) LC-MS: *m/z* calculated for C12H6Cl2N3O4SxNH3: [M + H]^+^, 374.97; found, 375.1

##### N‐(6‐methyl‐1,3‐benzothiazol‐2‐yl)‐5‐nitrothiophene‐2‐carboxamide (CBK277772)



1-Propanephosphonic acid cyclic anhydride (227 µL, 0.38 mmol) was added to a solution of 5-nitro-2-thiophenecarboxylic acid (25 mg, 0.16 mmol) in DMF (0.5 mL). After 30 min triethylamine (0.11 mL, 0.8 mmol) was added. After another 30 min 6‐methyl‐1,3‐benzothiazol-2-amine (20 mg, 0.12 µmol) was added and stirred overnight. MeOH (0.5 mL) was added and the volatile solvents were evaporated. Addition of 0.5–1 mL of MeOH and H_2_O (1–2 mL) and filtration and drying in vacuum gave the title compound as a brown solid. Yield 15 mg. ^1^H NMR (400 MHz, DMSO-*d*_6_) δ ppm 2.41 (s, 3 H) 7.29 (dd, *J* = 8.29, 1.18 Hz, 1 H) 7.57 (d, *J* = 8.21 Hz, 1 H) 7.77 (s, 1 H) 8.03 (br. s., 1 H) 8.17 (d, *J* = 4.26 Hz, 1 H) 12.79–14.19 (m, 1 H). LC-MS: *m/z* calculated for C13H10N3O3S2: [M + H]^+^, 320.01; found, 320.1

##### N‐(5-chloro-4-methyl‐1,3‐benzothiazol‐2‐yl)‐5‐ nitrothiophene‐2‐carboxamide (CBK277775)



1-Propanephosphonic acid cyclic anhydride (227 µL, 0.38 mmol) was added to a solution of 5-nitro-2-thiophenecarboxylic acid (25 mg, 0.16 mmol) in DMF (0.5 mL). After 30 min triethylamine (0.11 mL, 0.8 mmol) was added. After another 30 min 5-chloro-4‐methyl‐1,3‐benzothiazol-2-amine (25 mg, 0.12 µmol) was added and stirred overnight. MeOH (0.5 mL) was added and the volatile solvents were evaporated. Addition of 0.5–1 mL of MeOH and H_2_O (1-2 mL) and filtration and drying in vacuum gave the title compound as a brown solid. Yield 12 mg. ^1^H NMR (400 MHz, DMSO-*d*_6_) δ ppm 2.31 (s, 3 H) 7.54 (d, *J* = 8.53 Hz, 1 H) 7.71 (d, *J* = 8.78 Hz, 1 H) 8.03 (d, *J* = 4.27 Hz, 1 H) 8.23 (d, *J* = 4.27 Hz, 1 H) 10.77 (s, 1 H). LC-MS: *m/z* calculated for C13H8ClN3O3S: [M + NH4]^+^, 371.00; found, 371.0

##### N‐(4,6‐dichloro‐1,3‐benzothiazol‐2‐yl)‐5‐ nitrothiophene‐2‐carboxamide (CBK277776)



1-Propanephosphonic acid cyclic anhydride (227 µL, 0.38 mmol) was added to a solution of 5-nitro-2-thiophenecarboxylic acid (25 mg, 0.16 mmol) in DMF (0.5 mL). After 30 min triethylamine (0.11 mL, 0.8 mmol) was added. After another 30 min 4,6-dichloro-4-1,3‐benzothiazol-2-amine (25 mg, 0.11 µmol) was added and stirred overnight. MeOH (0.5 mL) was added and the volatile solvents were evaporated. Addition of 0.5–1 mL of MeOH and H_2_O (1-2 mL) and filtration and drying in vacuum gave the title compound as a brown solid. Yield 13 mg. ^1^H NMR (400 MHz, DMSO-*d*_6_) δ ppm 7.72 (d, *J* = 2.01 Hz, 1 H) 8.22 (d, *J* = 2.01 Hz, 1 H) 8.23 (d, *J* = 4.27 Hz, 1 H) 8.34 (d, *J* = 4.52 Hz, 1 H) 13.82 (br. s., 1 H). LC-MS: *m/z* calculated for C12H6Cl2N3O3S2: [M + H]^+^, 373.91; found, 374.0

##### N‐(6‐bromo‐1,3‐benzothiazol‐2‐yl)‐5‐nitrofuran‐2‐carboxamide (CBK277778)



1-Propanephosphonic acid cyclic anhydride (250 µL, 0.4 mmol) was added to a solution of 5-nitro-2-furanoic acid (32 mg, 0.2 mmol)) in DMF (1 mL). After 30 min triethylamine (125 µL, 0.9 mmol) was added. After another 30 min 6‐bromo‐1,3‐benzothiazol-2-amine (29 mg, 0.13 µmol) was added and stirred overnight. MeOH (1 mL) was added and the volatile solvents were evaporated. H_2_O was added and a solid was filtered off and dried in vacuum to afford the title compound as a brown solid. Yield 30 mg. ^1^H NMR (400 MHz, DMSO-*d*_6_) δ ppm 7.64 (dd, *J* = 8.53, 2.01 Hz, 1 H) 7.69 (br. s., 1 H) 7.84 (d, *J* = 3.76 Hz, 1 H) 7.88 (br. s., 1 H) 8.31 (s, 1 H) 13.56 (br. s., 1 H). LC-MS: *m/z* calculated for C12H7BrN3O4S: [M + H]^+^, 367.93; found, 368.0

##### N‐(5‐chloro‐4‐methyl‐1,3‐benzothiazol‐2‐yl)‐5‐nitrofuran‐2‐carboxamide (CBK277779)



1-Propanephosphonic acid cyclic anhydride (250 µL, 0.4 mmol) was added to a solution of 5-nitro-2-furanoic acid (25 mg, 0.16 mmol) in DMF (1 mL). After 30 min triethylamine (125 µL, 0.9 mmol) was added. After another 30 min 5‐chloro-4-methyl‐1,3‐benzothiazol-2-amine (26 mg, 0.13 µmol) was added and stirred overnight. MeOH (1 mL) was added and the volatile solvents were evaporated. H_2_O was added and a solid was filtered off and dried in vacuum to afford the title compound as a beige solid. Yield 9 mg. ^1^H NMR (400 MHz, DMSO-*d*_6_) δ ppm 7.52 (d, *J* = 8.53 Hz, 1 H) 7.63 (d, *J* = 4.02 Hz, 1 H) 7.71 (d, *J* = 8.53 Hz, 1 H) 7.84 (d, *J* = 4.02 Hz, 1 H) 10.74 (s, 1 H). LC-MS: *m/z* calculated for C13H8ClN3O4S: [M + NH4]^+^, 355.03; found, 355.1

##### 5‐nitro‐N‐(6‐nitro‐1,3‐benzothiazol‐2‐yl)furan‐2‐carboxamide (CBK277781)



1-Propanephosphonic acid cyclic anhydride (250 µL, 0.4 mmol) was added to a solution of 5-nitro-2-furanoic acid (25 mg, 0.16 mmol) in DMF (1 mL). After 30 min triethylamine (125 µL, 0.9 mmol) was added. After another 30 min 6‐nitro‐1,3‐benzothiazol-2-amine (25 mg, 0.13 µmol) was added and stirred overnight. MeOH (1 mL) was added and the volatile solvents were evaporated. H_2_O was added and a solid was filtered off and dried in vacuum to afford the title compound as a brown solid. Yield 25 mg. ^1^H NMR (400 MHz, DMSO-*d*_6_) δ ppm 7.85 (d, *J* = 4.02 Hz, 1 H) 7.88–7.98 (m, 2 H) 8.33 (dd, *J* = 8.91, 2.38 Hz, 1 H) 9.11 (d, *J* = 2.51 Hz, 1 H) 13.88 (br. s., 1 H). LC-MS: *m/z* calculated for C12H7N4O6S: [M + H]^+^, 335.00; found, 335.1

##### N‐(4,6‐dichloro‐1,3‐benzothiazol‐2‐yl)‐5‐nitrofuran‐2‐carboxamide (CBK277782)



1-Propanephosphonic acid cyclic anhydride (250 µL, 0.4 mmol) was added to a solution of 5-nitro-2-furanoic acid (32 mg, 0.2 mmol) in DMF (1 mL). After 30 min triethylamine (125 µL, 0.9 mmol) was added. After another 30 min 4,6‐dichloro‐1,3‐benzothiazol-2-amine (28 mg, 0.13 µmol) was added and stirred overnight. MeOH (1 mL) was added and the volatile solvents were evaporated. H_2_O was added and a solid was filtered off and dried in vacuum to afford the title compound as a brown solid. Yield 7 mg. ^1^H NMR (400 MHz, DMSO-*d*_6_) δ ppm 7.72 (d, *J* = 2.05 Hz, 1 H) 7.86 (d, *J* = 3.95 Hz, 1 H) 8.01 (d, *J* = 3.95 Hz, 1 H) 8.21 (d, *J* = 1.90 Hz, 1 H) 13.79 (br. s., 1 H). LC-MS: *m/z* calculated for C12H6Cl2N3O4S: [M + H]^+^, 357.94; found, 357.9

### Preparation of in vivo solutions

A Captisol solution was made by dissolving 4 g Captisol in H_2_O up to a final volume of 10 mL during gentle stirring for 30 min. Sodium 6‐ethoxy‐N‐(5‐nitrofuran‐2‐carbonyl)‐1,3‐benzothiazol‐2‐aminide (25 mg) was added and gentle stirring continued for 1 h. Tris buffer (2.5 mL, 25 mM, pH 8.3) was added and the solution was pH adjusted to ca pH = 7 with 1 M HCl. The solution was filtered through a 45 micron filter and kept at 4 °C.

Another solution (vehicle) was made as above with the exception that no Sodium 6‐ethoxy‐N‐(5‐nitrofuran‐2‐carbonyl)‐1,3‐benzothiazol‐2‐aminide was added.

#### Sodium 6‐ethoxy‐N‐(5‐nitrofuran‐2‐carbonyl)‐1,3‐benzothiazol‐2‐aminide (CBK291422N, sodium salt of CBK77)



Twenty milligrams of the solid was partly dissolved in EtOH and sodium hydroxide solution (5 × 10 µL, 2 M) was added to the mixture until the pH was basic and the color had changed to deep red-brown. By then the sodium salt has precipitated. The mixture was filtered and the solid washed with a little EtOH. Gave a dark violet solid. ^1^H NMR (400 MHz, DMSO-*d*_6_) δ ppm 1.30–1.37 (m, 3 H) 3.97–4.07 (m, 2 H) 6.83 (dd, *J* = 8.69, 2.69 Hz, 1 H) 7.16 (d, *J* = 3.79 Hz, 1 H) 7.28 (d, *J* = 2.53 Hz, 1 H) 7.35–7.41 (m, 1 H) 7.71 (d, *J* = 3.79 Hz, 1 H). ^13^C NMR (101 MHz, DMSO-*d*_6_) δ ppm 14.86 (s, 1 C) 63.41 (s, 1 C) 105.08 (s, 1 C) 113.40 (s, 1 C) 113.67 (s, 1 C) 114.31 (s, 1 C) 119.38 (s, 1 C) 134.31 (s, 1 C) 144.59 (s, 1 C) 150.77 (s, 1 C) 153.72 (s, 1 C) 156.32 (s, 1 C) 160.98 (s, 1 C) 166.75 (s, 1 C) LC-MS: *m/z* calculated for C14H12N3O5S: [M + H]^+^, 334.05; found, 334.1

## Results

### Cell-based, high-content screen for small molecule inhibitors of the UPS

**Fig. 1:**
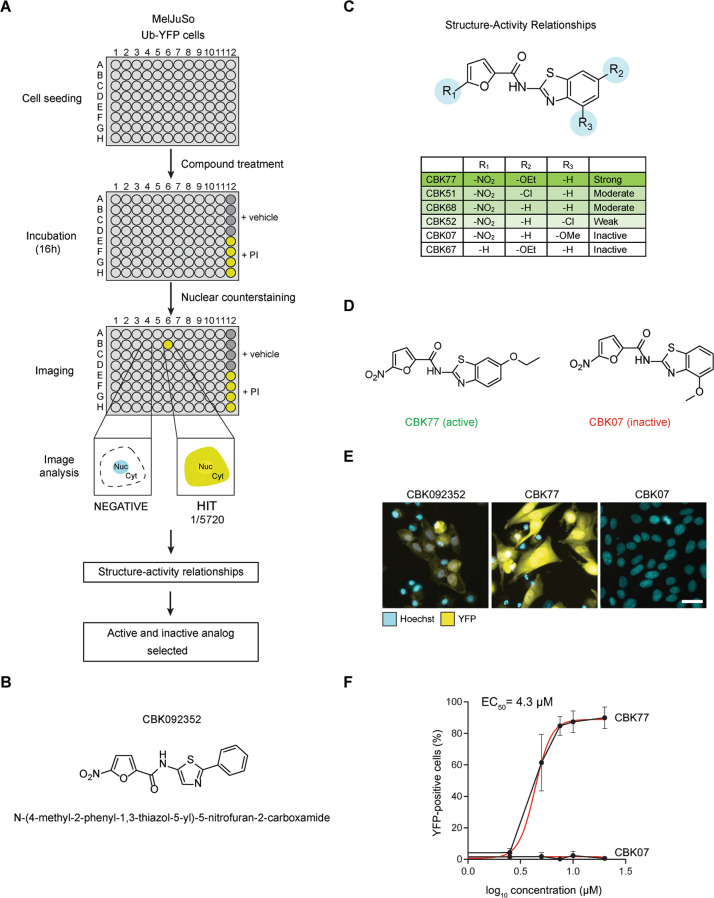
A cell-based phenotypic screen for inhibitors of the UPS. **A** Representation of the screen workflow. Positive hits were defined as compounds that accumulated the UFD reporter in the nucleus above basal levels, defined by the signal in the DMSO-treated wells (negative control). Positive control wells treated with epoxomicin (PI proteasome inhibitor, 100 nM) were also included in each plate of the screen. One positive hit was found in the initial screen, which formed the basis for a structure-activity relationship study (SAR). One active and one inactive structurally related compound were selected for further characterization. **B** Chemical structure of the hit compound CBK092352. **C** Summary of structure-activity relationships (SAR). Attenuation of activity is observed when R_3_ is substituted, whereas substituents in R_2_ are accepted. Removal of the nitro group results in an inactive compound. **D** Structures of the analogs selected for further characterization, the active compound CBK77 and the inactive compound CBK07. **E** Representative images of MelJuSo Ub-YFP cells treated for 16 h with the indicated compounds (10 µM). DMSO at 0.1% was used as negative control. The nuclei were counterstained with Hoechst and cells imaged live with an automated widefield microscope. Scale bar = 20 µm. **F** Concentration-response experiments performed with MelJuSo Ub-YFP cells. Cells were treated for 6 h with a range of compound concentrations. Nuclei were stained with Hoechst and cells were directly imaged live with an automated widefield microscope. Data are represented as mean ± SD of three independent experiments. Nonlinear curve fitting is depicted in red. The half-maximal effective concentration (EC_50_) upon CBK77 treatment is shown (4.3 µM, 95% confidence interval 3.8–5.0).

With the aim of finding novel UPS inhibitors, we performed a phenotypic screen for small molecules that cause a general accumulation of a UPS reporter substrate (Fig. 1A; Supplementary Table S1). We used a human melanoma cell line, MelJuSo, that stably expresses the UPS reporter ubiquitin^G76V^-yellow fluorescent protein (Ub-YFP) [20]. Cells were treated with a diverse set of 5720 small molecules from the Chemical Biology Consortium Sweden (www.cbcs.se) for 16 h at 10 µM. The screen resulted in the identification of the 1,3‐thiazol compound CBK092352 (Fig. 1B, Supplementary Fig. S1A), which caused strong accumulation of the Ub-YFP substrate as well as an increase in apoptotic nuclei (Supplementary Fig. S1B,C).

From the initial hit, we selected 41 analogs based on substructure and fingerprint similarity searches, as well as manual searches of the larger compound collection, to investigate a possible structure-activity relationship (SAR). This set of analogs was also further expanded through the synthesis of 19 structurally related compounds (Supplementary Fig. S2A; Supplementary Table S2). Most notably, SAR analysis showed that the 5-nitrofuran group is critical for biological activity of these compounds. Moreover, the O-ethoxy group at the 6 position (R_2_) improved the inhibitory effect of the compound as compared to the unsubstituted compound, whereas an O-methoxy group at the 4 position (R_3_) abrogated the effect (Fig. 1C). The importance of R_3_ was further strengthened by methyl and chlorine substitutions at this position, which also strongly reduced UPS inhibition and induction of cell death.

We selected a pair of closely related analogues of which one compound, CBK006377 (referred to as CBK77; N‐[6‐ethoxy‐1,3‐benzothiazol‐2‐yl]‐5‐nitrofuran‐2‐carboxamide), displayed profound UPS impairment and cellular toxicity, while the second compound, CBK085907 (referred to as CBK07; N‐(4‐methoxy‐1,3‐benzothiazol‐2‐yl)‐5‐nitrofuran‐2‐carboxamide), lacked these activities (Fig. 1D,E). The EC_50_ of CBK77 was determined as 4.3 µM (6 h treatment, 95% C.I 3.8–5.0 µM) with no detectable inhibition for CBK07 in the tested concentration range (Fig. 1F). It should, however, be mentioned that CBK07 is not completely inert as we observed modest UPS impairment and toxicity at high concentrations (>50 µM) over longer incubations (24 h) (Supplementary Fig. S2B). The uptake of CBK77 and CBK07 in cells was comparable, excluding that the strongly reduced activity of CBK07 could be attributed to a loss of cell permeability (Supplementary Fig. S2C). Together these data show that CBK77 blocks degradation of a reporter substrate of the UPS and induces cell death.

### CBK77 causes global impairment of the UPS

**Fig. 2:**
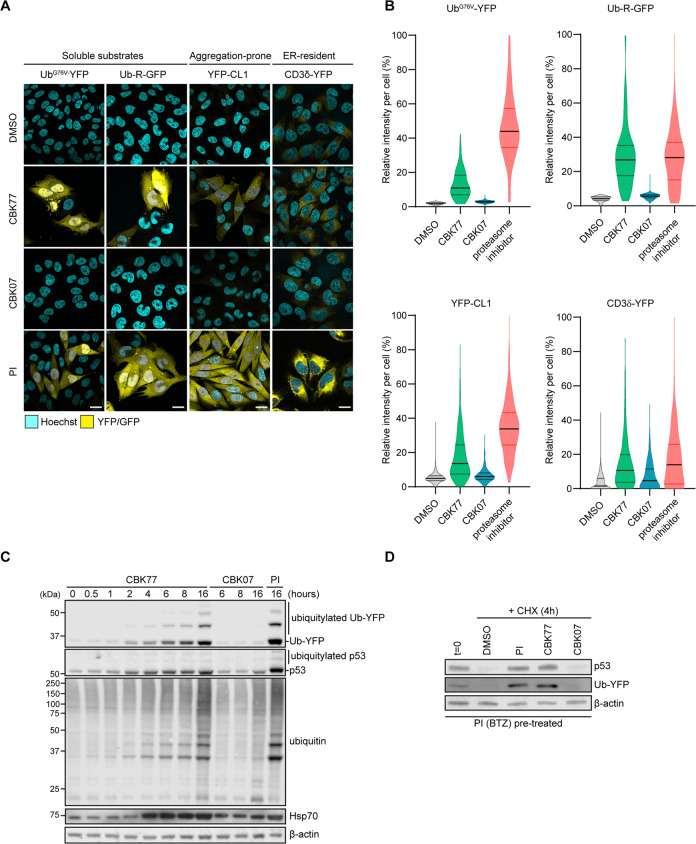
The UPS inhibitor CBK77 causes global impairment of the UPS. **A** Representative confocal images of MelJuSo cells stably expressing the indicated fluorescent UPS reporters. Cells were treated for 16 h with either CBK77 or CBK07 (10 µM) or bortezomib (PI proteasome inhibitor, 25 nM) as a positive control. DMSO at 0.1% was used as negative control. Cells were fixed and nuclei were counterstained with Hoechst. Scale bars = 20 µm. **B** Cells were treated for 16 h with CBK77 or CBK07 (10 µM for all reporter cell lines besides YFP-CL1, treated at 5 µM). The proteasome inhibitor bortezomib (25 nM) and DMSO 0.1% were included as positive and negative controls, respectively. After nuclei counterstaining with Hoechst, cells were imaged in an automated manner with a widefield fluorescent microscope. Frequency and distribution of the reporter (YFP) intensity per cell are shown as violin plots from a representative experiment (of two independent experiments). Data are normalized to percentages were 0% = minimum value in the DMSO sample; 100% = maximum value in the proteasome inhibitor sample. Black lines within each distribution represent the median; colored lines represent the upper and lower interquartile range limits. Ub-YFP: *n* = 591, *n* = 283, *n* = 514, *n* = 337; Ub-R-GFP: *n* = 391, *n* = 190, *n* = 201, *n* = 208; YFP-CL1: *n* = 666, *n* = 470, *n* = 623, *n* = 397; CD3δ-YFP: *n* = 717, *n* = 335, *n* = 505, *n* = 447 in DMSO, CBK77, CBK07, and bortezomib-treated cells, respectively. **C** MelJuSo Ub-YFP cells were treated with either CBK77, CBK07 (10 µM) or epoxomicin (PI = proteasome inhibitor, 100 nM) and harvested at the indicated timepoints. Cell lysates were analyzed by immunoblotting with the indicated antibodies. Representative blots from one out of three independent experiments are shown. **D** MelJuSo Ub-YFP cells were pretreated for 3 h with the reversible proteasome inhibitor bortezomib (BTZ, 25 nM) to increase the levels of Ub-YFP and p53 before the chase. Samples were taken directly after pretreatment (t = 0). The remaining wells were co-treated with cycloheximide (CHX, 50 µg/ml) and the indicated compounds and harvested after 4 h (CHX 4 h). Cell lysates were analyzed by immunoblotting with the indicated antibodies. Representative blots from one of two independent experiments are shown.

The effect of CBK77 was analyzed on additional reporters that represent different classes of proteasome substrates: ubiquitin-arginine-GFP (Ub-R-GFP), a soluble N-end rule substrate; YFP-CL1, an aggregation-prone substrate and lastly, a reporter based on the T cell receptor subunit CD3δ (CD3δ-YFP), which is degraded by ER-associated degradation (ERAD) [21]. Administration of CBK77, but not CBK07, resulted in accumulation of each of these substrates (Fig. 2A, B). Interestingly, the level of inhibition varied between the different classes of substrates: the N-end rule substrate accumulated to the same level as observed with proteasome inhibitor, whereas the other three substrates accumulated to intermediate levels. This suggests that CBK77 has a molecular mechanism that is distinct from proteasome inhibitors.

Western blot analysis showed an increase in the levels of Ub-YFP as well as the endogenous UPS substrate p53 already 2 h after administration of CBK77 in contrast to CBK07 (Fig. 2C). Several high-molecular weight products were detected in both the Ub-YFP and p53 immunoblots, which gave rise to a ladder pattern that is characteristic for ubiquitin-modified proteins (Fig. 2C). Moreover, CBK77 caused a general accumulation of ubiquitin conjugates with similar kinetics as observed for polyubiquitylated Ub-YFP and p53 (Fig. 2C). Notably, the accumulation of ubiquitylated p53 suggests that UPS impairment is responsible for the accumulation of p53 and not functional stabilization as part of the apoptotic response, which is typically regulated by inhibiting ubiquitylation of p53 [22]. At the same time, a contribution of functional stabilization of p53 cannot be excluded. In line with a general inhibition of ubiquitin-dependent degradation, CBK77 treatment increased the levels of Hsp70, which is a common stress response elicited by inhibition of protein degradation [23] (Fig. 2C). Analysis of the turnover of Ub-YFP and p53 upon inhibition of protein synthesis with cycloheximide showed that the accumulation of these substrates was due to a delay in their clearance (Fig. 2D). Together, these data show that CBK77 inhibits proteasomal degradation resulting in accumulation of polyubiquitylated substrates.

### CBK77 causes irreversible UPS impairment followed by caspase-dependent cell death

**Fig. 3:**
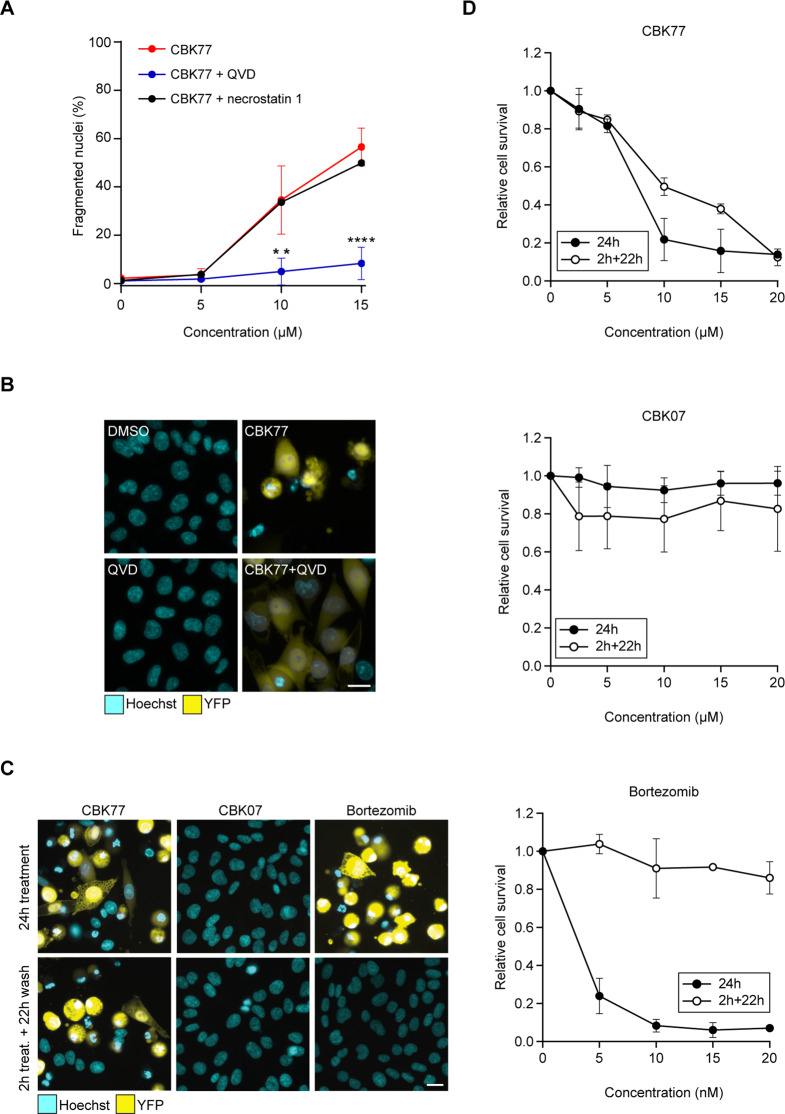
CBK77 causes irreversible UPS impairment followed by caspase-dependent cell death. **A** MelJuSo Ub-YFP cells were treated with either 5, 10, or 15 µM of CBK77 with or without co-treatment with the pan-caspase inhibitor Q-VD-OPh (QVD, 20 µM) or necrostatin 1 (30 µM) for 24 h. DMSO-treated cells (0.2 or 0.3%, respectively) were used as negative control. After incubation cells were fixed, counterstained with Hoechst and imaged with an automated widefield microscope. The number of apoptotic cells was determined in an automated manner based on nuclear staining intensity and fragmentation. Data are shown as mean percentage of apoptotic cells ±SD from three independent experiments. ** adjusted *p* ≤ 0.01, **** adjusted *p* ≤ 0.0001 (two-way ANOVA with Dunnett’s multiple comparisons test). **B** Representative images from **A**. Scale bar = 20 µm. **C** Representative images from washout experiments performed in MelJuSo Ub-YFP cells. Cells were either continuously treated for 24 h (“24 treatment”), or treated for 2 h with DMSO, CBK77 (10 µM) or the reversible proteasome inhibitor bortezomib (25 nM) and further incubated with inhibitor-free medium up to 24 h (“2 h treat. + 22 h wash”). Nuclei were stained with Hoechst. Imaging was then performed live with an automated widefield microscope. Scale bar = 20 µm. **D** Quantification of **C**. The cell count was obtained based on the nuclei staining. Data are shown as mean relative cell count to DMSO-treated cells ±SD from three technical replicates of a representative experiment of three independent experiments.

The UPS is critical for cell viability and blockade of the pathway typically results in induction of apoptosis [24]. Consistent with apoptotic cell death, administration of the pan-caspase inhibitor Q-VD-OPh significantly reduced cell death of CBK77-treated cells, in contrast to the compound necrostatin, which prevents necroptosis, a form of nonapoptotic cell death (Fig. 3A). It is noteworthy that the pan-caspase inhibitor did not prevent accumulation of the reporter substrate, in line with the model that induction of apoptosis is a consequence and not a cause of UPS impairment (Fig. 3B).

Interestingly, CBK77 has an irreversible effect on the functionality of the UPS as a 2 h treatment of cells with CBK77 followed by a washout and 22 h incubation in the absence of the compound still caused a comparable level of UPS inhibition to that observed in cells that had been continuously incubated with CBK77 for 24 h, in contrast to results for the reversible proteasome inhibitor bortezomib (Fig. 3C). Accordingly, transient exposure to CBK77 was sufficient to induce cell death whereas cells were able to recover from a short incubation with bortezomib (Fig. 3D). Altogether, this shows that CBK77 induces irreversible inhibition of proteasomal degradation followed by caspase-dependent apoptosis.

### CBK77 does not cause global inhibition of proteasomal or DUB activity

**Fig. 4:**
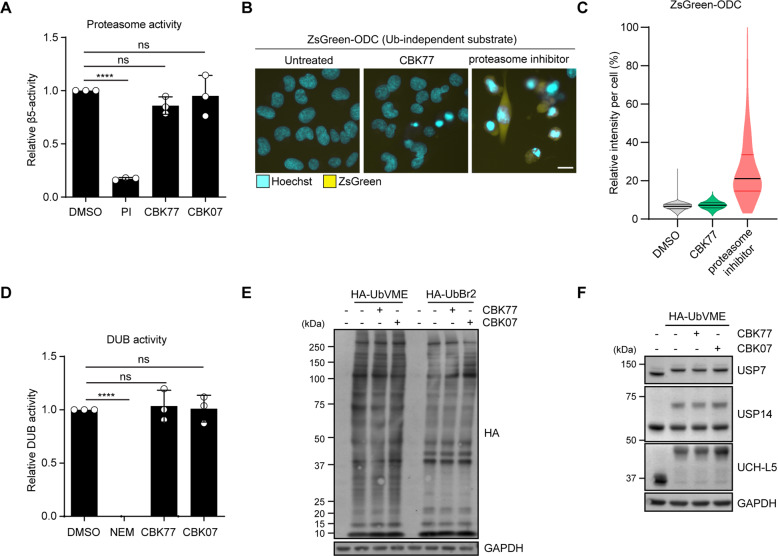
CBK77 does not cause global inhibition of proteasomal or DUB activity. **A** MelJuSo parental cells were treated with DMSO 0.1%, CBK77 10 µM, CBK07 10 µM or epoxomicin (PI proteasome inhibitor, 200 nM) for 4 h. The chymotrypsin activity (β5 subunit) of the proteasome was assessed in the corresponding lysates by following conversion of the fluorogenic Suc-LLVY-AMC substrate over 1 h. Data are shown as mean ± SD of three independent experiments. ns nonsignificant. **** adjusted *p*-value  (One-way ANOVA with Dunnett’s multiple comparisons test). Suc-LLVY-AMC Suc-Leu-Leu-Val-Tyr-7-amido-4-methylcoumarin. **B** Representative images of MelJuSo ZsGreen-ODC cells. Cells were treated for 16 h with CBK77 (5 µM). The proteasome inhibitor epoxomicin (100 nM) was included as positive control. Nuclei were counterstained with Hoechst and the cells imaged live with an automated widefield microscope. Scale bar = 20 µm. **C** Quantification of **B**. The nuclear YFP intensity per cell was quantified using Cell Profiler. Frequency and distribution of the measured YFP intensity per cell are shown as violin plots. Data are normalized to percentages were 0% = minimum value in the DMSO sample; 100% = maximum value in the proteasome inhibitor sample. *n* > 500 cells (DMSO); *n* = 250 cells (CBK77); and *n* = 298 cells (proteasome inhibitor) from a representative experiment (of two independent experiments). Black lines within each distribution represent the median; colored lines represent the upper and lower interquartile range limits. **D** MelJuSo Ub-YFP cells were treated with DMSO 0.1%, CBK77 10 µM or CBK07 10 µM for 4 h. The global deubiquitylating activity in the cells was assessed in the corresponding lysates by following the conversion of the fluorogenic ubiquitin-AMC substrate over 30 min. A sample containing DMSO-treated cell lysate + 15 mM NEM was included as a negative control. Data are shown as mean ± SD of three independent experiments. ns nonsignificant. **** adjusted *p*-value  (One-way ANOVA with Dunnett’s multiple comparisons test). AMC 7-amido-4-methylcoumarin, DUB deubiquitylating enzyme, NEM N-ethylmaleimide. **E** MelJuSo parental cells were treated with DMSO 0.1%, CBK77 10 µM, or CBK07 10 µM for 6 h. The enzymatic activity of DUBs was assessed by incubating the cell extracts with the DUB-specific probes HA-UbVME and HA-UbBr2. A sample was incubated without probe as a technical control for DUB labelling. DUB activity was assessed by SDS-PAGE and western blotting with an anti-HA antibody. **F** The same samples displayed in **E** were probed with antibodies against the specified enzymes. Active DUBs shift upwards in the gel due to the incorporation of the probe.

Proteasome activity and DUB activity are two primary targets of compounds that have been reported to cause a general impairment of the UPS [5, 18]. Using an in vitro enzymatic assay, we analyzed the effect of CBK77 treatment on the chymotrypsin-like activity of the proteasome, which is the primary catalytic site and the main target of clinically used proteasome inhibitors [5]. In vitro analysis of the chymotrypsin-like activity in lysates of CBK77-treated cells showed that CBK77 did not inhibit this activity (Fig. 4A). Moreover, CBK77 did not impair the degradation of the ubiquitin-independent reporter ZsGreen ornithine decarboxylase (ODC) [13] (Fig. 4B, C), further excluding that CBK77 impairs UPS activity at the level of the proteasome.

A role for inhibition of deubiquitylating (DUB) enzymes in the mode of action of CBK77 is unlikely as our analysis showed that CBK77 did not cause an overall reduction in DUB activity in cell lysates (Fig. 4D). Moreover, profiling of DUB activity with activity probes [25] did not reveal apparent differences in the levels or activity of DUBs in lysates from cells treated with CBK77 (Fig. 4E). There was also no effect of CBK77 on the activity on USP7 [26, 27], USP14 [28], and UCH-L5 [6], three DUBs that we considered of specific interest as these have been found to be targeted by other experimental compounds (Fig. 4F). Thus, CBK77 causes a global impairment of ubiquitin-dependent proteasomal degradation without global inhibition of proteasome or DUB activity.

### NAD(P)H:quinone oxidoreductase 1 (NQO1) is critical for the CBK77-mediated UPS impairment

**Fig. 5:**
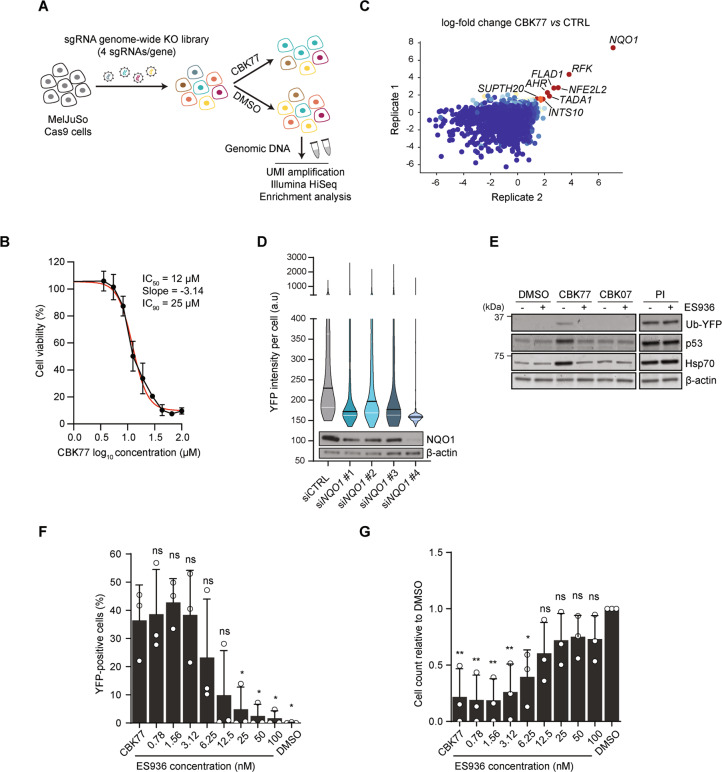
NAD(P)H:quinone oxidoreductase 1 (NQO1) is a critical factor for CBK77-mediated UPS impairment. **A** Schematic drawing depicting the pooled CRISPR–Cas9 screening paradigm. MelJuSo cells expressing Cas9 were infected with a lentiviral sgRNA library (four sgRNAs per gene). Cells were treated with DMSO (0.25%) or CBK77 25 µM three times during a 5-day period. The resulting cell populations were harvested and subjected to barcode sequencing and analysis. Two technical replicates were performed. **B** Dose-response experiments performed with MelJuSo Ub-YFP cells. Cell viability was assessed after 72 h. Data are represented as mean ± SD of three independent experiments. Nonlinear curve fitting is depicted in red. The half-maximal inhibitory concentration (IC_50_) and slope of the curve are used to calculate the theoretical IC_90_ concentration. **C** Scatter plot of the average gene log-fold change (LFC) in CBK77-treated cells versus control (CTRL)-treated cells in the two replicates as calculated using MaGeCK [17]. Each dot represents a gene and is colored from lower (blue) to higher (red) LFC values. The names of genes with an average LFC > 1.5 are shown. **D** MelJuSo Ub-YFP cells were depleted of NQO1 for 48 h with the indicated siRNAs (10 nM) and then treated for 24 h with CBK77 (5 µM). Nontargeting siRNA (siCTRL) was used as control. After the treatment, nuclei were counterstained with Hoechst and cells imaged live with a widefield automated microscope. Frequency and distribution of the nuclear YFP intensity per cell are shown as violin plots from one experiment (of two independent experiments). Black lines within each distribution represent the median; white lines represent the upper and lower interquartile range limits. The knockdown efficiencies are shown in the blot below. **E** MelJuSo Ub-YFP cells were co-treated with either DMSO, CBK77, CBK07 (10 µM) or bortezomib (PI proteasome inhibitor, 25 nM) with or without the NQO1 inhibitor ES936 (100 nM) for 6 h. Cell lysates were analyzed by immunoblotting with the indicated antibodies. Representative blots from one of three independent experiments are shown. **F** MelJuSo Ub-YFP cells were treated with CBK77 (10 µM) alone or in combination with the NQO1 inhibitor ES936 at the indicated concentrations for 24 h. After the treatment, cells were fixed and nuclei counterstained with Hoechst. Cells were imaged with an automated widefield microscope. The percentage of YFP-positive cells are shown as mean of three independent experiments. Error bars depict the SEM. ns nonsignificant, * adjusted *p* ≤ 0.05 (one-way ANOVA with Dunnett’s multiple comparisons test). **G** The cell count from **F** was obtained based on the nuclei staining. Data are shown as mean of three independent experiments. Error bars depict the SEM. ns nonsignificant, * adjusted *p* ≤ 0.05, ** adjusted *p* ≤ 0.01 (one-way ANOVA with Dunnett’s multiple comparisons test).

To gain further insight in the actual mode of action of CBK77, we performed an unbiased, genome-wide CRISPR interference screen to identify mechanisms of resistance by exposing MelJuSo cells three times to the IC_90_ concentration of CBK77 (25 µM) (Fig. 5A, B). We found a clear enrichment of guides targeting the oxidoreductase NQO1 in the CBK77-treated population (Fig. 5C, Supplementary Table S3). Moreover, the gene encoding for Nrf2 (*NFE2L2*), a transcriptional stimulator of NQO1 [29], as well as the gene encoding for flavin adenine dinucleotide synthase 1 (*FLAD1*), which is responsible for synthesis of the NQO1 coenzyme flavin adenine dinucleotide (FAD) [30], were highly enriched in the CBK77-resistant cell population (Fig. 5C, Supplementary Table S3).

The enrichment of these candidates suggested that the presence of NQO1 may be critical for the mode of action of CBK77. In line with this hypothesis, we found that cellular depletion of NQO1 using siRNAs reduced the accumulation of the reporter substrate Ub-YFP in response to CBK77 treatment (Fig. 5D). The catalytic activity of NQO1 was required for the inhibitory effect of CBK77 as ES936, a potent irreversible inhibitor of NQO1 activity [31], abrogated the CBK77-induced accumulation of Ub-YFP, p53, and Hsp70 (Fig. 5E). Moreover, administration of ES936 caused a concentration-dependent decrease in CBK77-induced UPS impairment (Fig. 5F) and improved cell survival (Fig. 5G). Inhibition of ALDH-2, which has been reported to reduce 5-nitrofurans leading to general cell toxicity [32], did not alleviate the effects of CBK77 (Supplementary Fig. 3A, B).

### CBK77 is an NQO1 substrate

**Fig. 6:**
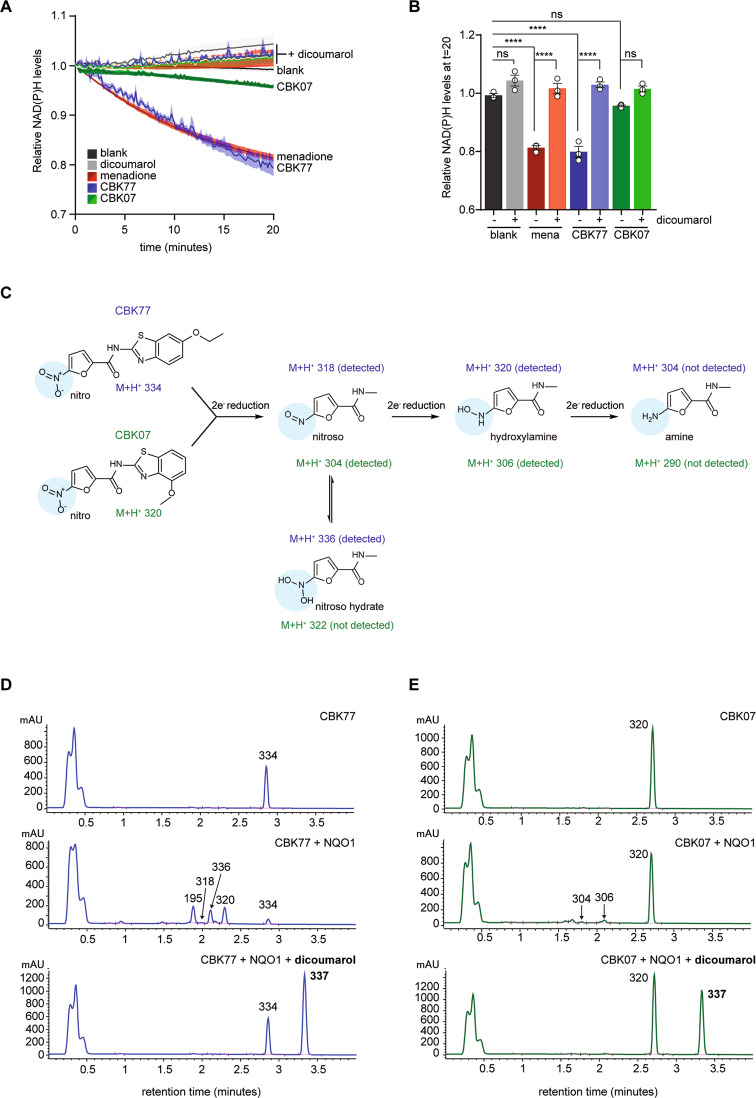
CBK77 is a substrate of NQO1. **A** Recombinant NQO1 was incubated with CBK77 or CBK07 (100 µM) in the presence or absence of the NQO1 inhibitor dicoumarol (100 µM). The NQO1 substrate menadione (40 μM) with or without dicoumarol was used as control. Plot showing the NAD(P)H levels over time, which are reduced due to the conversion of NAD(P)H to NADP + in the presence of NQO1 substrates. Data are shown as average NAD(P)H levels ±SEM (shadowed area) of three independent experiments. **B** Bar plot of the values displayed in the last timepoint (t = 20 min) in **H**. Datapoints are shown as aligned dots ±SEM. ns nonsignificant, **** adjusted *p* ≤ 0.0001 (one-way ANOVA with Tukey’s multiple comparisons test). **C** Schematic drawing depicting sequential two-electron reductions of the nitro group present in CBK77 and CBK07. These expected sequential reductions produce amines via nitroso and hydroxylamine intermediates. The impact of these reductions on the expected M + H^+^ after electrospray ionization (ESI+) of the compounds are shown below each structure in a color-coded manner (blue = related to CBK77; green = related to CBK07). **D** HPLC-MS DAD (305 ± 90 nm) chromatograms of in vitro reactions incubating CBK77 (100 µM) with or without recombinant NQO1. A sample with dicoumarol (100 µM) was included to assess dependency on NQO1’s activity. The M + H^+^ of expected metabolites found after electrospray ionization (ESI+) in the MSD1 report are annotated above each peak. **E** HPLC-MS DAD (305 ± 90 nm) chromatograms of in vitro reactions incubating CBK07 (100 µM) with or without recombinant NQO1. A sample with dicoumarol (100 µM) was included to assess dependency on NQO1’s activity. The M + H^+^ of expected metabolites found after electrospray ionization (ESI+) in the MSD1 report are annotated above each peak.

Even though the natural substrates of NQO1 are quinones, NQO1 can also reduce certain nitroaromatic compounds [33, 34]. To test if CBK77 is metabolized by NQO1, we performed an in vitro enzymatic assay that measures NAD(P)H consumption by NQO1 when reducing substrates. Strikingly, CBK77 induced the oxidation of NAD(P)H comparably to the genuine quinone substrate menadione, which suggests that CBK77 is efficiently metabolized by NQO1 (Fig. 6A, B). Accordingly, the enzymatic reaction towards CBK77 was blocked by the NQO1 inhibitor dicoumarol. On the contrary, NQO1 showed very limited enzymatic activity towards the inactive CBK07 (Fig. 6A, B).

The two-electron reduction of nitroaromatic compounds, which has been previously described [35], is expected to proceed stepwise involving the formation of three major species: the nitroso, hydroxylamine and amine derivates (Fig. 6C). Analytical HPLC-MS confirmed that the level of CBK77 (M + H^+^ 334) in the in vitro reaction rapidly decreased in the presence of NQO1, which was accompanied by the appearance of the 5-nitrosamine derivative (M + H^+^ 318) and the hydroxylamine derivative (M + H^+^ 320), corresponding with two- and four-electron reductions of the parental compound, respectively (Fig. 6D). Other species detected corresponded with the hydrate form of the 5-nitrosamine derivative (M + H^+^ 336) and the 6-ethoxy-1,3-benzothiazol-2-amine (M + H^+^ 195), which appears to be the product from hydrolysing the amide of the parent compound (Fig. 6D). CBK07 was poorly metabolized, giving rise to trace amounts of the 5-nitrosamine derivative (M + H^+^ 304) and hydroxylamine derivative (M + H^+^ 306) (Fig. 6E). The appearance of the CBK77 and CBK07 derivatives could be blocked by the addition of the NQO1 inhibitor dicoumarol (Fig. 6D,E). Overall, these data confirm that CBK77 is efficiently metabolized by NQO1, resulting in the production of metabolites that are in line with the anticipated stepwise two-electron reductions of the parental compound.

### CBK77 interacts with ubiquitin in an NQO1-dependent fashion

**Fig. 7:**
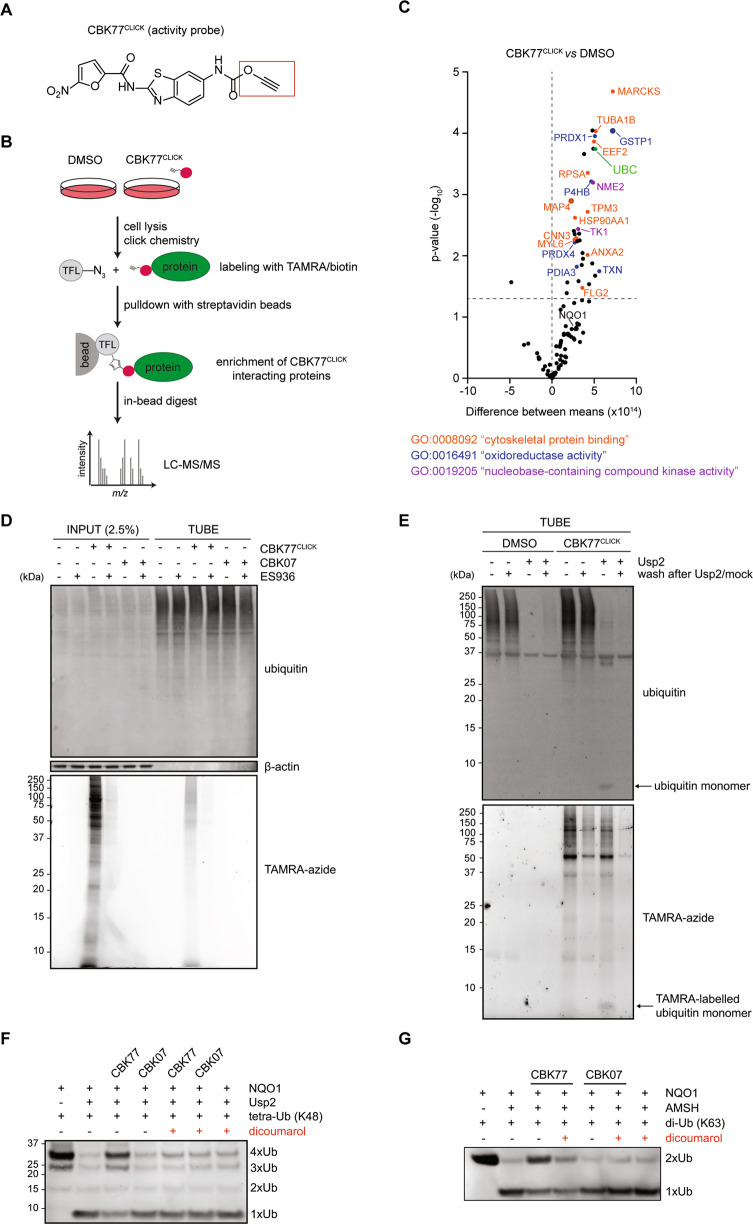
CBK77 interacts with ubiquitin in an NQO1-dependent fashion. **A** Chemical structure of activity probe CBK77^CLICK^. Boxed in red is the alkyne moiety, which can be used for covalently linking tags via *click* chemistry. **B** Schematic drawing representing the chemical proteomics workflow. **C** Volcano plot depicting the results of multiple *t*-testing in the protein abundance in CBK77^CLICK^-treated cells versus DMSO. Abundance is defined by the average peak area of the three most intense peptides, including unique and non-unique peptides. The *x*-axis represents the difference between means; the *y*-axis represents the statistical significance as the −log_10_ *p*-value. Dashed lines indicate the threshold for significance (*p*-value ≤ 0.05) and the point with no difference between the groups (x = 0). No correction for multiple comparisons was carried out to have a less stringent, hypothesis-generating approach. The names of the proteins involved in significantly enriched molecular functions are colored according to the legend. **D** MelJuSo cells were treated with either DMSO, CBK77, CBK77^CLICK^ (100 µM), or CBK77^CLICK^ in combination with the NQO1 inhibitor ES936 (100 nM) for 45 min. After cell lysis and labelling of the compound with a TAMRA-azide fluorophore, ubiquitin was pulled down with TUBE-agarose beads. Binding of the compound to ubiquitin was assessed via TAMRA detection and immunoblotting with a ubiquitin antibody. Representative images from one of three independent experiments are shown. **E** MelJuSo Ub-YFP cells were treated with CBK77^CLICK^ (10 µM) for 1 h. After cell lysis and labelling of the compound with a TAMRA-azide fluorophore, ubiquitin was pulled down with TUBE-agarose beads. After incubation, the beads were treated with or without Usp2 for 1 h. A set of samples were stopped directly with addition of LDS-sample buffer, while a second set was further washed to remove unbound proteins. Binding of the compound to ubiquitin was assessed via TAMRA detection and immunoblotting with a ubiquitin antibody. Representative images from one of four independent experiments are shown. **F** Recombinant NQO1 was incubated with CBK77 or CBK07 (100 µM) in the presence or absence of the NQO1 inhibitor dicoumarol (100 µM) in the presence of K48-linked tetraubiquitin chains. Samples were then treated with the recombinant catalytic domain of the DUB Usp2 and cleavage of the ubiquitin moieties was assessed via SDS-PAGE and immunoblotting with a ubiquitin antibody. Representative blot from one of three independent experiments is shown. **G** Recombinant NQO1 was incubated with CBK77 or CBK07 (100 µM) with K63-linked di-ubiquitin chains in the presence or absence of the NQO1 inhibitor dicoumarol (100 µM). Samples were then treated with 233 nM of recombinant AMSH and cleavage of the ubiquitin moieties was assessed via SDS-PAGE and immunoblotting with a ubiquitin antibody. Representative blot from one of two independent experiments is shown.

To identify molecular targets of CBK77, we developed an activity-based probe that allowed labelling of interacting molecules [36]. For this purpose, we took advantage of our SAR analysis and generated the probe CBK77^CLICK^ for activity-based target identification by introducing an alkyne group in the 6 position of the 1,3-benzothiazol group (Fig. 7A). We confirmed that the introduction of the terminal alkyne group did not affect CBK77 activity (Supplementary Fig. 4A, B). The introduced alkyne moiety allows covalent linkage of a tag-of-choice via copper(I)-catalyzed azide alkyne cycloaddition (CuAAC) [37].

For identification of the CBK77 interactome, we prepared cell lysates from vehicle or CBK77^CLICK^-treated cells and introduced the trifunctional linker (TFL) TAMRA-azide-biotin for fluorescent visualization (TAMRA fluorophore) and biochemical enrichment (biotin group) (Fig. 7B). In-gel detection of streptavidin-bead eluates confirmed efficient enrichment of CBK77^CLICK^ conjugates (Supplementary Fig. 4C). Mass spectrometry-based analysis identified 40 proteins to be enriched in streptavidin pull-downs of CBK77 ^CLICK^-treated samples (Fig. 7C). Upon gene ontology analysis, one of the CBK77^CLICK^-enriched classes consisted of proteins with oxidoreductase activity, recapitulating the involvement of a redox-related toxicity mechanism found in the CRISPR/Cas9 screen (Supplementary Table S4). NQO1 was identified but not significantly enriched, consistent with NQO1 being important for processing of the compound but not a primary target of activated CBK77. Most notably, ubiquitin (UBC) was identified as one of the most enriched proteins in the pulldown with the CBK77^CLICK^ probe (Fig. 7C).

In siRNA-mediated depletion of the proteins that were enriched in the CBK77 interactome, we did not observe accumulation of Ub-YFP for any of the candidates (Supplementary Fig. 4D). Not surprisingly, the only CBK77-interacting protein that upon depletion phenocopied CBK77-induced UPS impairment was ubiquitin itself (Supplementary Fig. 4E). To validate the covalent modification of endogenous ubiquitin by CBK77, we performed ubiquitin pull-downs with tandem-repeated ubiquitin-binding entities (TUBEs) [38]. A specific smear of high-molecular weight CBK77^CLICK^-labelled proteins was detected that was reminiscent to the signal observed for endogenous ubiquitin conjugates, which was strongly reduced by ES936 (Fig. 7D, Supplementary Fig. 4F).

The labelling of ubiquitin conjugates could be due to covalent binding of CBK77 to ubiquitylated substrates or, alternatively, to ubiquitin itself. To address this question, we performed again enrichment of ubiquitin conjugates with TUBEs but treated the pulled down conjugates with the ubiquitin specific protease 2 (Usp2) to disassemble the ubiquitin chains. Treatment with Usp2 resulted in the appearance of TAMRA-labelled ubiquitin monomers, indicating that ubiquitin is modified by CBK77 (Fig. 7E). Washing of the beads after Usp2 treatment resulted in the disappearance of the ubiquitin monomers, in line with the specificity of the TUBEs for polyubiquitin chains, while some of the TAMRA-labelled smear remained bound to TUBEs. This suggest that the remaining signal is, at least in part, due to TAMRA-labelled polyubiquitylated substrates that were not deconjugated by Usp2.

### CBK77-modified ubiquitin conjugates resist deubiquitylating enzymes

Even though CBK77 does not affect overall DUB activity, it may nevertheless inhibit deubiquitylation by rendering the ubiquitin chains less susceptible to DUB activity. K48-linked tetraubiquitin chains that had been exposed to NQO1-activated CBK77 were less efficiently disassembled by Usp2 in vitro, which could be prevented by blocking NQO1 activity with dicoumarol (Fig. 7F). An in vitro assay with K63-linked ubiquitin chains and the deubiquitylating enzyme AMSH, a metalloprotease representing a different class of deubiquitylating enzymes with a preference for K63-linked ubiquitin chains [39], revealed a similar inhibitory effect (Fig. 7G). Our data suggest that CBK77-modified ubiquitin chains are less susceptible to deubiquitylating activities in vitro, providing a plausible explanation for the selective inhibition of ubiquitin-dependent proteasomal degradation by CBK77.

### CBK77 reduces growth of NQO1-proficient human cancer cells and xenograft tumors in mice

**Fig. 8:**
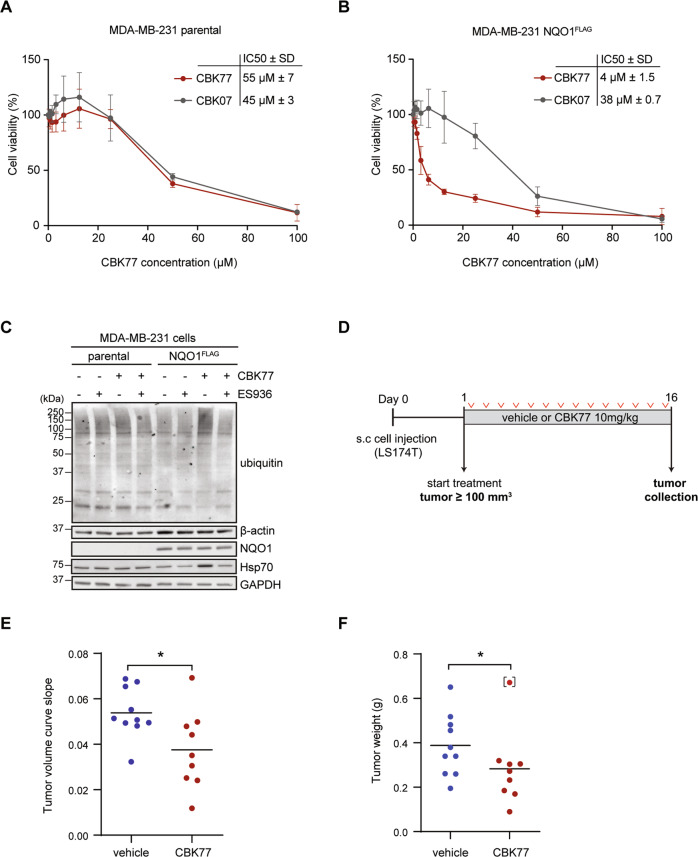
CBK77 reduces growth of NQO1-proficient human cancer cells and xenograft tumors in mice. **A** Concentration-response experiments performed with MDA-MB-231 parental cells. Cell viability was assessed after 72 h using the WST-1 proliferation reagent. Data are represented as mean ± SEM of three independent experiments. The half-maximal inhibitory concentrations (IC_50_) ± SD are shown. **B** Concentration-response experiments performed with MDA-MB-231 cells stably expressing NQO1^FLAG^. Cell viability was assessed after 72 h using the WST-1 proliferation reagent. Data are represented as mean ± SEM of three independent experiments. The half-maximal inhibitory concentrations (IC_50_) ± SD are shown. **C** MDA-MB-231 parental and NQO1^FLAG^-expressing cells were incubated with the indicated compounds for 6 h. Lysates were probed with the indicated antibodies. Representative blots from one of three independent experiments are shown. **D** Experimental timeline for the xenotransplant experiment. **E** Scatter plot of the tumor volume curve slopes obtained in a simple linear regression model (according to ref. [19]). *p-value = 0.024 (two-sided, unpaired *t*-test). **F** Scatter plot of the tumor weights (grams) at necropsy (day 16). The median value is depicted as a solid black line. A Grubbs’ outlier test identified one tumor as a significant outlier in the CBK77 group (marked in brackets). Note that all tumors in the treated group, besides the one identified as an outlier, are clustered below the median of the vehicle group. *p-value = 0.014 (two-sided, unpaired *t*-test, excluding outlier datapoint).

To further explore the importance of NQO1 for potential antitumor activity of CBK77, we took advantage of a naturally occurring NQO1 polymorphism. Individuals that express the unstable NQO1^C609T^ variant have undetectable or trace levels of the NQO1 protein and therefore very low NQO1 activity [40]. As expected, a breast cancer cell line derived from an individual expressing this NQO1 variant (MDA-MB-231) was largely resistant to CBK77 with an IC_50_ of >50 µM, which was very similar to the sensitivity of the cell line to the poorly active CBK07 analogue (Fig. 8A). However, ectopic expression of stable NQO1 in MDA-MB-231 cells resulted in 10-fold increased sensitivity to CBK77 (Fig. 8B). Ectopic expression of stable NQO1 in MDA-MB-231 cells resulted in the accumulation of ubiquitin conjugates and an increase in Hsp70 levels, which was abrogated by ES936, showing that the proposed anticancer effect of CBK77 is dependent on NQO1 activity (Fig. 8C).

For an in vivo assessment of the efficacy of CBK77 against tumors, we selected the human colon adenocarcinoma cell line LS174T, which expresses functional NQO1 at a comparable level to the MelJuSo cell line used in the high-content screen (Supplementary Fig. 5A). Accordingly, LS174T cells were found to be sensitive to CBK77 (Supplementary Fig. 5B). LS174T cells were injected subcutaneously into the flanks of immunocompromised NMRI nu/nu mice. When the tumors had reached a minimum size of 0.1 mL, mice were randomly assigned to either daily intraperitoneal administration of vehicle or 10 mg/kg CBK77 (Fig. 8D). During treatment, we did not observe weight loss or obvious behavioural signs of toxicity (Suppl. Fig. 5C). Consistent with its in vitro activity, we found that CBK77 administration resulted in a significant reduction in tumor growth rates (Fig. 8E) and tumor weights at necropsy (Fig. 8F). These data show that CBK77 inhibits growth of an NQO1-proficient cell line in a xenotransplant tumor model without overt signs of toxicity.

## Discussion

An attractive strategy for enhancing tumor specificity is the use of prodrugs that are activated in the altered intracellular environment of cancer cells. It has been shown that the elevated levels of NQO1 can provide a means to restrict the effect of redox-activated prodrugs primarily to cancer cells [41]. A notable example of an NQO1-activatable antitumorigenic drug is the naturally occurring quinone Mitomycin C, which upon reduction by NQO1 behaves as a DNA alkylating agent [42].

NQO1 has been linked to processes that involve the UPS. Notably, apo-NQO1 interacts and is in vitro degraded by the 20 S proteasome [43]. Moreover, NQO1 inhibits proteasomal degradation of ODC [44], p53 [45], and HIF1α [32]. The tight link between NQO1 and the UPS and its ability to interact with the proteasome may be of relevance for the inhibitory mechanism as this may position the short-lived, reactive CBK77 metabolites in close proximity to key players of the UPS, such as ubiquitin.

Our data suggest that CBK77 targets a protein centrally involved in ubiquitin-dependent proteasomal degradation. In line with this notion, we identified ubiquitin as a protein that is covalently modified by the activity probe. Moreover, CBK77 impaired the cleavage of ubiquitin chains in vitro of DUB enzymes of different families, supporting a model in which impaired disassembly of polyubiquitin chains leads to an accumulation of UPS substrates that causes cell death. Blocking of the UPS by ubiquitin-interacting compounds is not unprecedented. Ubistatins are a group of large molecules that non-covalently interact with two ubiquitin monomers simultaneously, thereby interfering with the targeting of polyubiquitylated proteins to the proteasome [10]. Future work focusing on the interaction between CBK77 and ubiquitin, and to what extent CBK77-modified ubiquitin conjugates contribute to UPS impairment and cell death, will be required to clarify CBK77’s mechanism of action.

Although CBK77 passed PAINS filters [46], the structural alert moiety nitrofuran raised some medicinal chemistry concerns. It should be noted, however, that the 5-nitrofuran compound Nitrofurantoin has been used for treatment of urinary tract infection with relatively mild adverse effects [47]. Moreover, another nitrofuran-based compound, Nifurtimox, is used as a trypanocidal drug for treatment of Chagas disease [48]. In our testing of CBK77 in a xenotransplant mouse model, we did not observe acute signs of toxicity caused by CBK77 while the tumor growth was reduced. It is noteworthy that in an earlier high-throughput screen, related nitrofuran compounds were found to activate the heat-shock response by an unknown mechanism [49]. Our data provide an explanation for this earlier observation, as it is well established that induction of the heat-shock response can be a direct consequence of a disturbed protein homeostasis [23]. Overall, we propose that CBK77 can provide a starting point for the development of a novel class of bioactivatable UPS inhibitors.

## Supplementary information

Supplementary Material

Supplementary Table S3

## Data Availability

The mass spectrometry proteomics data have been deposited to the ProteomeXchange Consortium via the PRIDE partner repository with the dataset identifier PXD019519. All the raw data, plasmids, and cell lines generated in this manuscript are available upon reasonable request. Further information and requests should be addressed to the corresponding author, NPD (nico.dantuma@ki.se).
