# Supplementary material for: Inhibition of the ubiquitin-proteasome system by an NQO1-activatable compound
Source: Cell Death Dis. 2021 Oct 6;12(10):914. doi: 10.1038/s41419-021-04191-9 (PMC8494907; doi:10.1038/s41419-021-04191-9)
Supplement: Supplementary file 1 — Supplementary Material [file 41419_2021_4191_MOESM1_ESM.docx]

**Supplementary Material**

**Figure S1.** Screen for UPS inhibitors.

**Figure S2.** CBK77 and CBK07 are both taken up by cells while only CBK77 efficiently blocks UPS activity.

**Figure S3.** ALDH-2 is not involved in bioactivation of CBK77.

**Figure S4.** Characterization of the activity probe CBK77^CLICK^ and related experiments to Fig. 7.

**Figure S5.** *In vivo* treatment with CBK77 does not have an effect on mouse weight.

**Supplementary Table S1.** Table summarizing the features of the high-content screen for small-molecule inhibitors of the UPS.

**Supplementary Table S2 (including S2.1, S2.2 and S2.3).** Tables summarizing the results of the secondary screen with 41 analogs to the hit compound CBK092352 and the expanded SAR with synthesized compounds.

**Supplementary Table S3**. Excelsheet showing the results of the two replicates performed in the CRISPR/Cas9 screen, summarized per gene.

**Supplementary Table S4.** Enriched molecular function terms in the network of proteins enriched upon CBK77 treatment in the proteomics dataset.

**Supplementary Table S5.** siRNAs used in this study.

**Supplementary Figure S1**


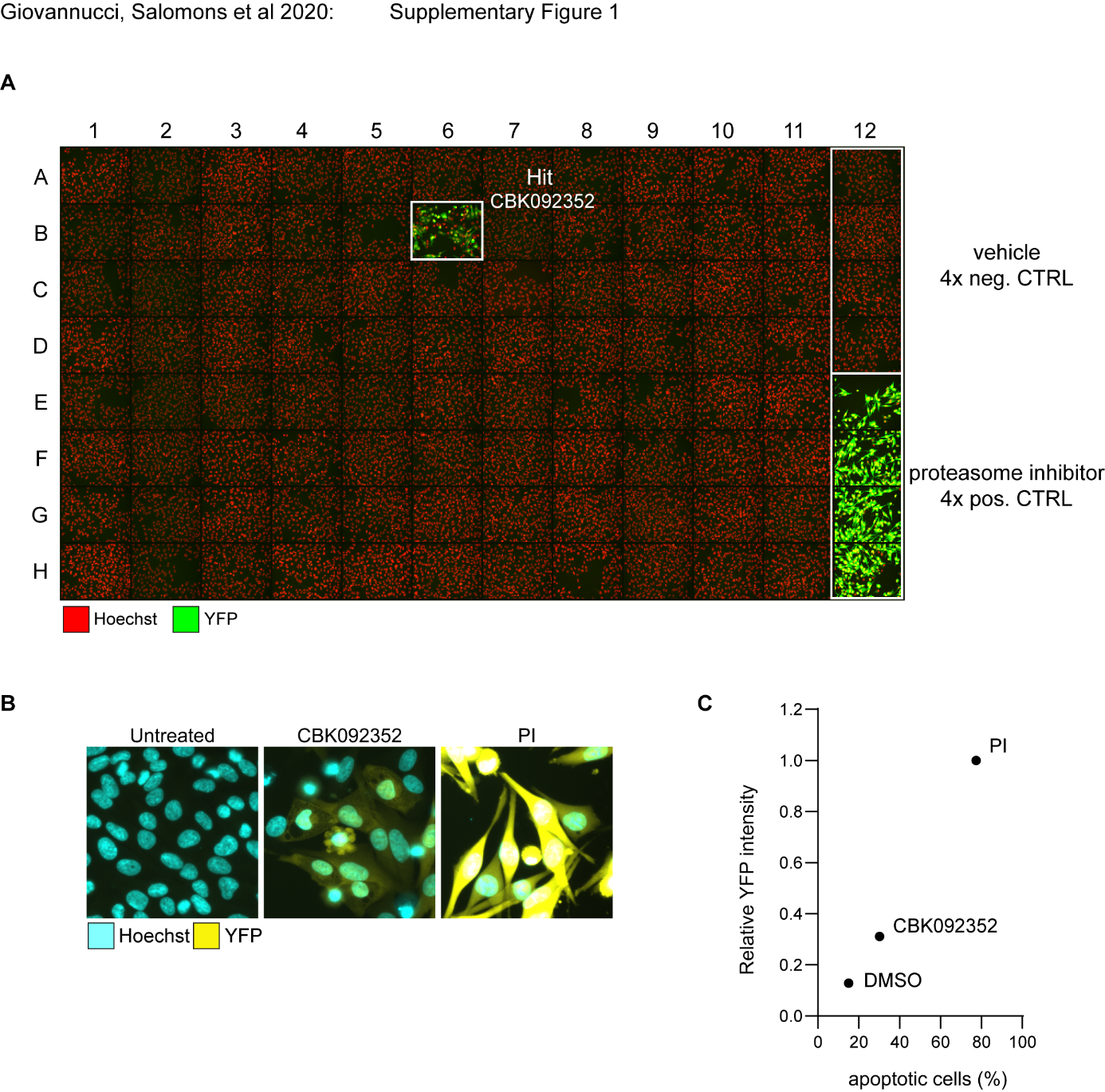


**Figure S1. Screen for UPS inhibitors.**

1. Overview of plate #35 from the screen, in which the active compound was found in position B6 (hit compound CBK092352).
2. Representative images of MelJuSo Ub-YFP cells from the indicated conditions in the screen plate #35.
3. Scatter plot showing the percentage of apoptotic cells (identified as fragmented nuclei based on the Hoechst staining) and the average YFP-intensity in the nucleus relative to the intensity in epoxomicin (proteasome inhibitor, PI) treated cells (relative YFP-intensity) from one experiment.

**Supplementary Figure S2**


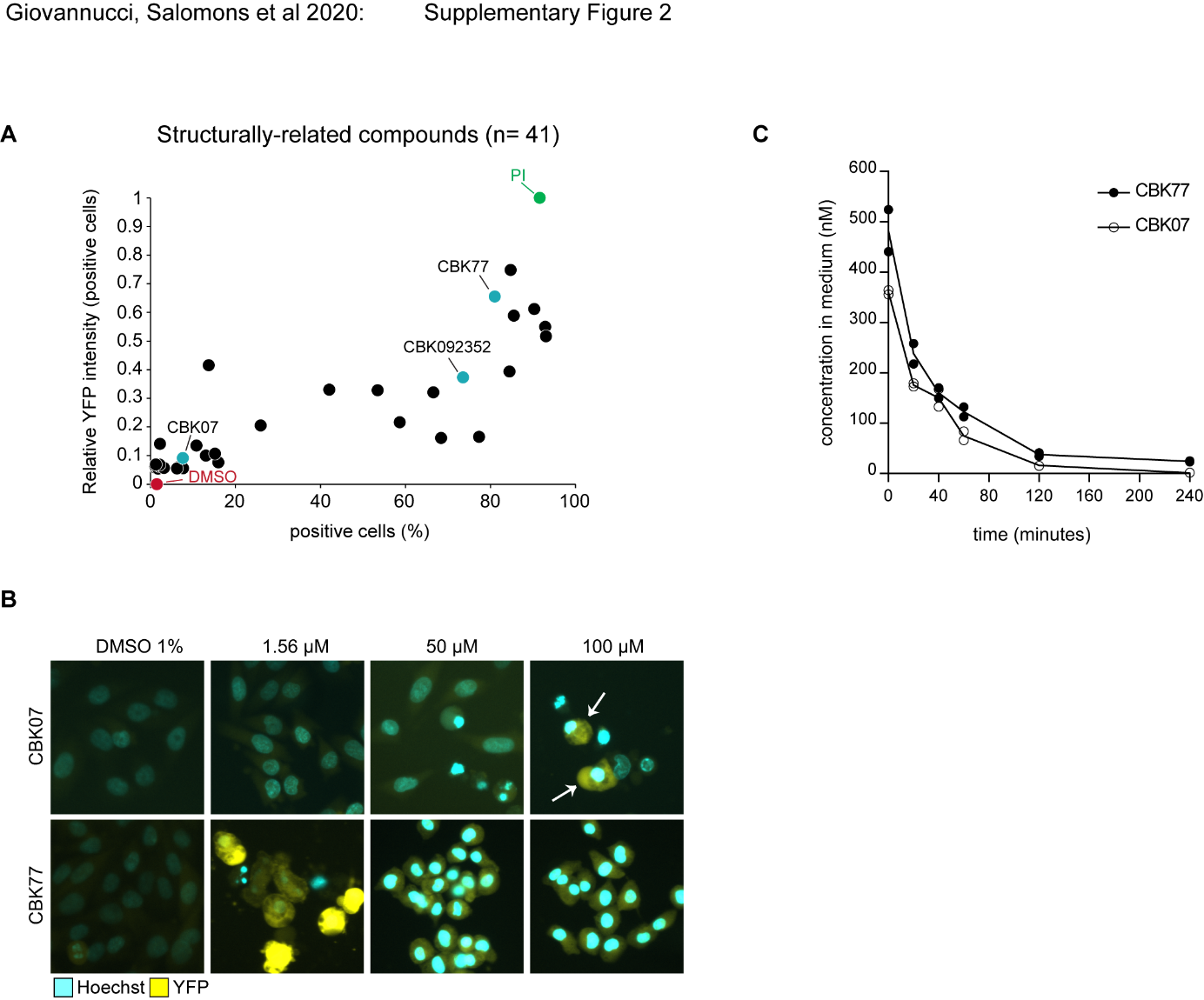


**Figure S2. CBK77 and CBK07 are both taken up by cells while only CBK77 efficiently blocks UPS activity.**

1. MelJuSo Ub-YFP cells were treated for 16 hours with selected compounds related to the benzothiazole hit molecule (10 µM). Epoxomicin (proteasome inhibitor = PI, 100 nM) was included as a positive control. After the treatment, nuclei were counterstained with Hoechst and cells were imaged live with a widefield automated microscope. The nuclear YFP intensity per cell was quantified using MetaXpress. Data are shown as the normalized YFP intensity to epoxomicin versus the percentage of positive cells in one experiment. Data can be found in Supplementary Table S2.
2. Representative images of MelJuSo Ub-YFP cells treated for 24 hours with CBK77 or CBK07 at the indicated concentrations. Nuclei were counterstained with Hoechst and after fixation cells imaged with an automated widefield microscope.
3. MelJuSo parental cells were treated with 1 µM of CBK77 or CBK07 for 20, 40, 60, 120 and 240 minutes. After incubation, the medium was removed and analyzed by mass spectrometry to calculate the amount of compound left in the medium. In parallel, the content of the cells was solubilized with a 60:40 acetonitrile:H_2_O mixture (50 mM acetonitrile final concentration) and analyzed by mass spectrometry to calculate the amount of compound inside the cells. The compound could not be detected in this latter analysis, probably due to a fast metabolism-related reaction, or to covalent binding to a target. The experiment was performed once.

**Supplementary Figure S3**


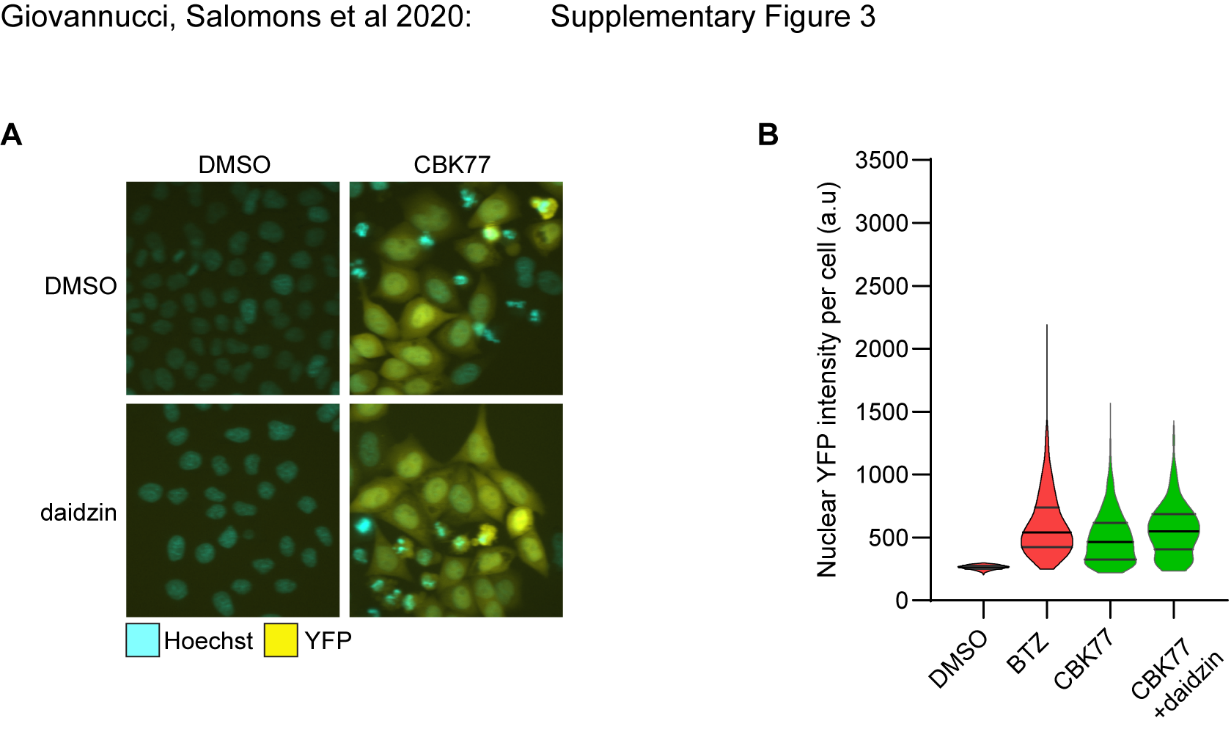


**Figure S3. ALDH-2 is not involved in bioactivation of CBK77.**

1. Representative images of HeLa Ub-YFP cells treated for 24 hours with DMSO 0.2% or daidzin 100 µM with or without co-treatment with CBK77 20 µM. Nuclei were counterstained with Hoechst and cells were imaged with an automated widefield microscope.
2. Quantification of (**A**). The nuclear YFP intensity per cell was quantified using the MetaXpress software. Frequency and distribution of the measured YFP intensity per cell are shown as violin plots. n= 1798 cells (DMSO); n = 509 cells (bortezomib); n = 912 cells (CBK77) and n= 493 cells (CBK77+daidzin) from a representative experiment (out of two independent experiments). Black lines within each distribution represent the median and the upper and lower interquartile range limits.

**Supplementary Figure S4**

**
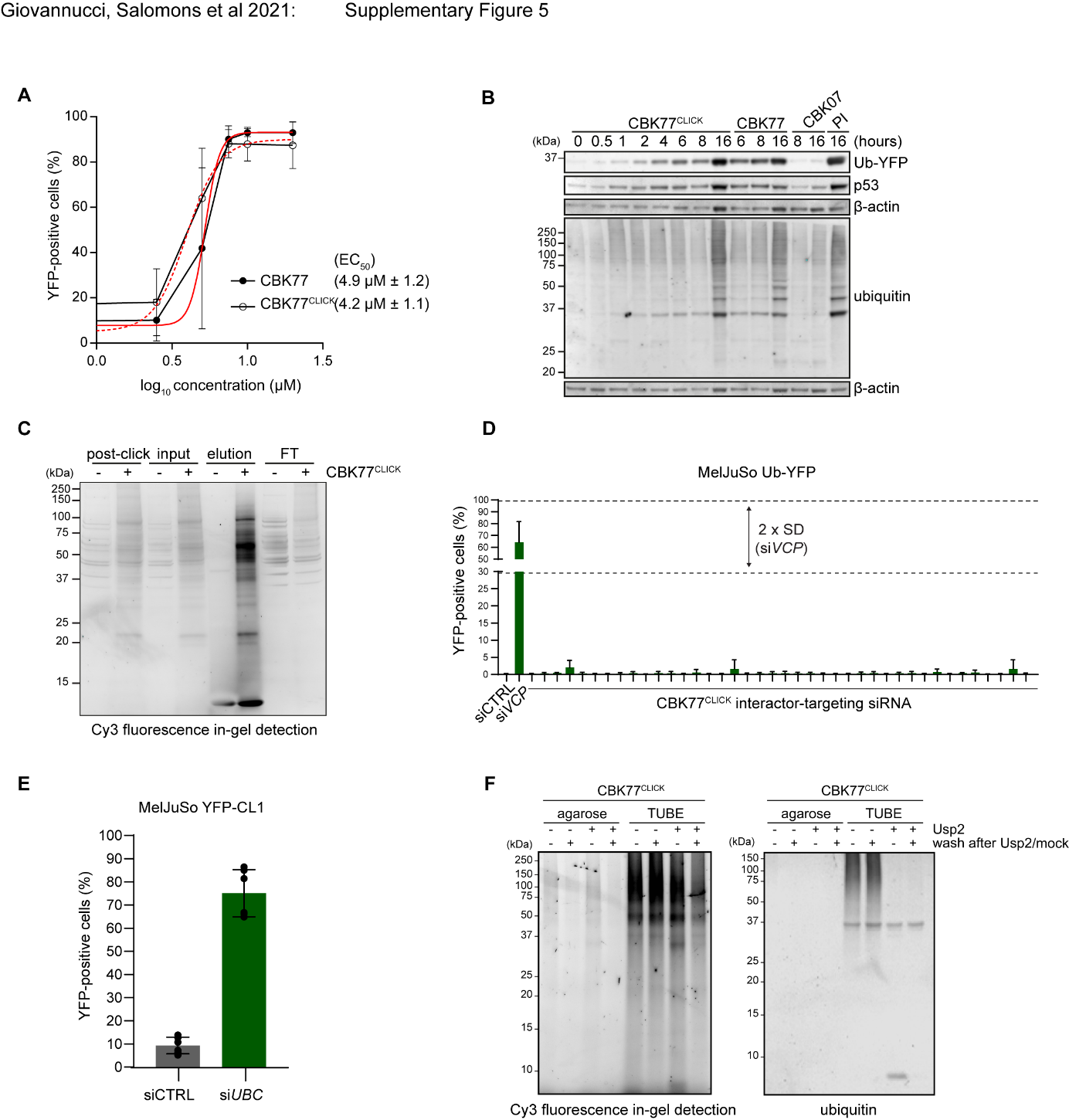
**

**Figure S4. Characterization of the activity probe CBK77^CLICK^ and related experiments to Fig. 7.**

1. Concentration-response experiments performed with MelJuSo Ub-YFP cells. Cells were treated for 6 hours with a range of compound concentrations. Nuclei were stained with Hoechst 33342 and cells directly imaged live with an automated widefield microscope. The number of cells accumulating the UFD reporter was quantified using the MetaXpress software. Data are represented as mean ± SD of three independent experiments. Non-linear curve fitting is depicted in red. The effective concentration (EC) at which 50% of the cells accumulate the UFD reporter above the basal threshold (EC_50_) upon CBK77^CLICK^ treatment is shown (4.2 µM).
2. MelJuSo Ub-YFP cells were treated with either CBK77^CLICK^, CBK77, CBK07 (10 µM) or epoxomicin (proteasome inhibitor = PI, 100 nM) and harvested at the indicated timepoints. Cell lysates were analyzed by immunoblotting with the indicated antibodies.
3. MelJuSo parental cells were treated with CBK77^CLICK^ (20 µM) for 1 hour. After labelling of the compound with the trifunctional linker TAMRA-azide-biotin, the labelled compound was pulled down with streptavidin beads.
4. siRNA subscreen with target genes selected from the CBK77^CLICK^ interactome mass spectrometry dataset. MelJuSo Ub-YFP cells were transfected with the indicated siRNAs for 48 hours. Nuclei were counterstained with Hoechst and after fixation cells imaged with an automated widefield microscope. Data are shown as mean percentage of YFP-positive cells ± SD of three independent experiments. siCTRL and the ubiquitin-selective segregase VCP/p97 (si*VCP*), which is critical for the degradation of Ub-YFP, were used as negative and positive controls, respectively.
5. MelJuSo YFP-CL1 cells were depleted of ubiquitin for 24 hours with the indicated siRNAs (si*UBC*, 30 nM). Non-targeting siRNA (siCTRL) was used as control. After the treatment, nuclei were counterstained with Hoechst and cells were imaged live in a widefield automated microscope. The average percentage of YFP-positive cells in the data pooled from two independent experiments (with three technical replicates each, shown as aligned dots) ± SD is shown as a bar plot.
6. MelJuSo Ub-YFP cells were treated with CBK77^CLICK^ (10 µM) for 1 hour. After cell lysis and labelling of the compound with a TAMRA-azide fluorophore, ubiquitin was pulled down with either control agarose beads or TUBE-agarose beads. After incubation, the beads were treated with or without Usp2 for 1 hour. A set of samples were stopped directly with addition of LDS-sample buffer, while a second set was further washed to remove unbound proteins. Binding of the compound to ubiquitin was assessed via TAMRA detection and immunoblotting with a ubiquitin antibody.

**Supplementary Figure S5**


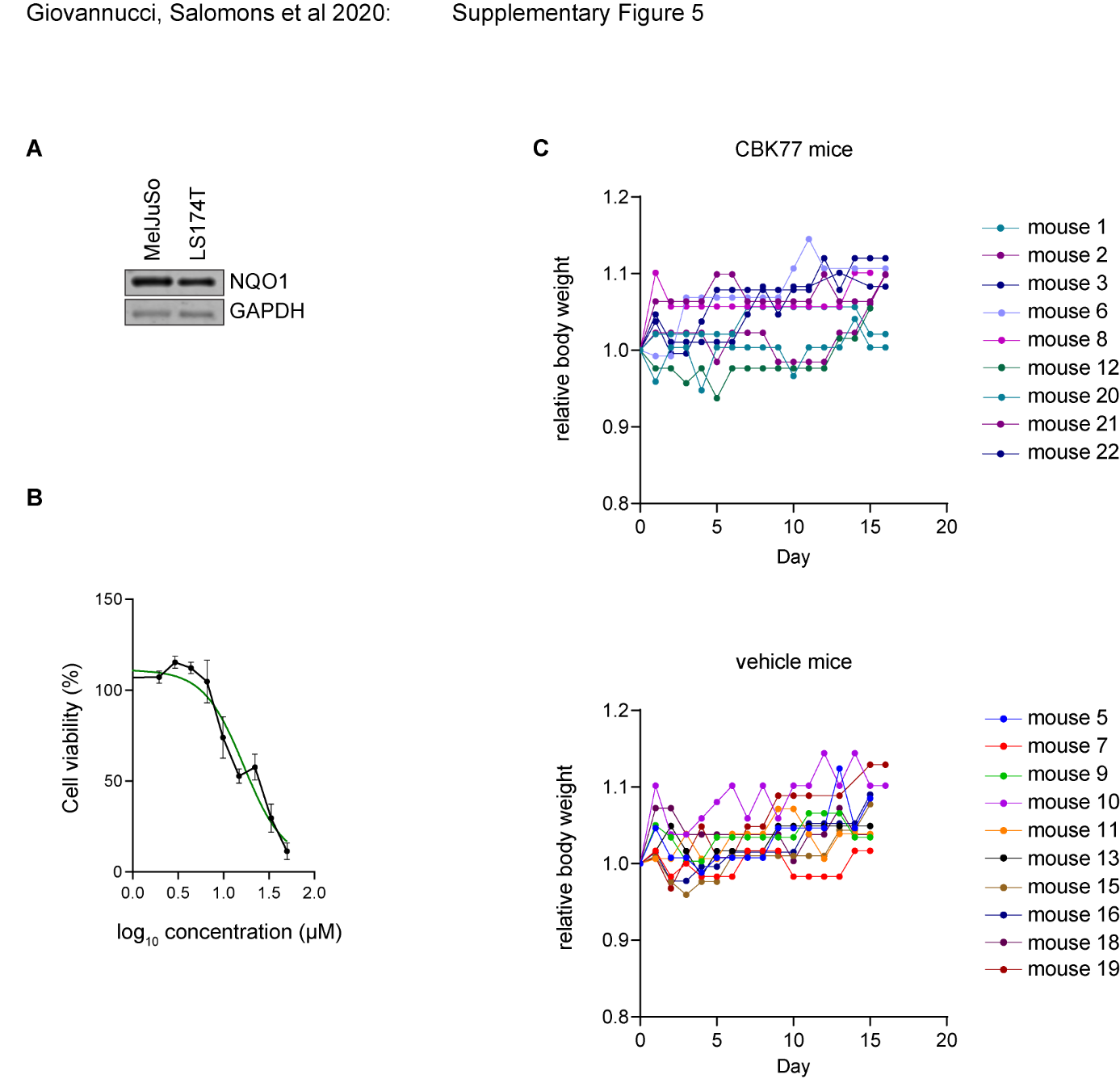


**Figure S5. *In vivo* treatment with CBK77.**

1. Western blot analysis of cell lysates from MelJuSo and LS174T cells comparing the levels of NQO1. GAPDH is shown as a loading control.
2. Concentration-response experiments performed with LS174T cells. Cell viability was assessed after 72 hours using the WST-1 proliferation reagent. Data are represented as mean ± SEM of five independent experiments. A curve-fit is shown in green.
3. Mice were weighed every second day after subcutaneous injection of LS174T cells until necropsy. Weights were normalized to the weight on the day of subcutaneous cell injection.

**Supplementary Table S1.**

| **Category** | **Parameter** | **Description** |
| --- | --- | --- |
| Assay | Type of assay | Phenotypic, cell-based high-content screen |
|  | Target | Ubiquitin-proteasome system (UPS) |
|  | Primary measurement | Fluorescence: accumulation of the UPS reporter Ub^G76V^-YFP (Ub-YFP in short) correlates with UPS activity. |
|  | Key reagents | MelJuSo Ub-YFP cell line; clear-bottom, black PS plates with tissue culture treated surface for imaging (Costar, 3904) RPMI 1640 media (Invitrogen, 21875) supplemented with 50 units/ml penicillin-streptomycin and 10% fetal bovine serum Hoechst 33342 nuclear stain (Life Technologies, H3570). |
|  | Assay protocol | 24 hours prior to screening with the chemical library a total of two 75 ml flasks of MelJuSo Ub-YFP stable cell lines were trypsinized after the cells had just reached confluency. Cells were mixed to produce a homogenous suspended cell solution and 90 µl of the suspension was seeded in each well of 96-well plates and incubated until compound addition. For each compound 2 µl at 10 mM concentration was added to 198 µl of RPMI1640 cell media at room temperature in 96 well plates. 10 µl of diluted compound was then transferred to the 90 µl of cells resulting in a final 1:1000 dilution of compounds and a total volume of 100 µl per well. Compounds were transferred to cell plates using an eight-tip pipette. 25 minutes before reaching 16 hours incubation, cells were stained with 20 µl solution of Hoechst/well (2 µg/ml Hoechst stain) and then analyzed with high-throughput microscopy. |
|  | Additional comments | 16 hours compound treatment time was originally selected by previous trials to determine time where the maximum fluorescent signal strength was obtained with cell line treated with positive control, epoxomicin (100 nM). |
| Library | Library size | 5720 compounds (diversity set) |
|  | Library composition | Compounds included in the primary screening set were selected to represent a diverse selection of a larger set of 65,000 compounds, while keeping a certain depth to allow crude structure–activity relationship studies. The selection was also biased towards lead-like and drug-like profiles with regards to molecular weight, hydrogen bond donors/acceptors and LogP. |
|  | Source | Most of these compounds were donated by Biovitrum AB and originate from both in-house and commercial sources (e.g, a set of approved drugs from Prestwick) |
|  | Additional comments | Compound stock solutions at 10 mM in DMSO were stored frozen at approximately -20°C in individual capped tubes in REMP 96 storage tube racks. The racks are stored in a REMP Small-Size Store, which allows cherry picking while the solutions are still frozen to minimize repetitive freeze-thaw cycles. |
| Screen | Format | 96-well |
|  | Concentration(s) tested | 10 µM |
|  | Plate controls | DMSO 0.1% (negative control); epoxomicin 100 nM (positive control, proteasome inhibitor) |
|  | Reagent/ compound dispensing system | Manual |
|  | Detection instrument and software | ImageXpress Micro (Molecular Devices; San Jose, CA, USA) coupled to MetaXpress software (Molecular Devices). |
|  | Assay validation/QC | z-prime of the assay (**z’ = 0.86**) based on quadruplicates of DMSO and epoxomicin controls in each plate of the screen following the formula: 1-($\frac{3x \left( SD positive control \right)+3x (SD negative control)}{(mean positive control-mean negative control)}$). For this calculation, the YFP intensity in each cell nucleus per image was calculated in an automated manner using an ImageJ macro. From these single-cell values, the average YFP nuclear intensity per image was calculated (sum of all YFP nuclear intensities divided by the number of nuclei). Finally, we calculated the average of all the DMSO images, and the average of epoxomicin images, and used these values for the formula. |
| Screen analysis | Hit criteria | Accumulation of the UPS reporter Ub-YFP using the threshold = (mean YFP intensity of the negative control + 3*SD of the negative control) |
|  | Hit rate | 1/5720 |
|  | Additional assay(s) | Cell death assay based on chromatin condensation and nuclear fragmentation. |
|  | Confirmation of hit purity and structure | High-performance liquid chromatography coupled to mass spectrometry (HPLC–MS). The estimated purity was calculated based on the integrated area for the expected mass compared with the areas of all other peaks. The result was manually controlled and if there were deviations from the expected outcome a meticulous investigation of the UV-response and MS was performed.  Structure was assessed by 1H NMR for known compounds and 1H and 13C NMR for all newly synthesized compounds |

**Supplementary Table S1.** High-content screen for small molecule inhibitors of the UPS.

**Supplementary Table S2**

| Compound | Chemical structure | Percentage of YFP-positive cells (%) | Relative nuclear YFP intensity (in positive cells) |
| --- | --- | --- | --- |
| CBK092352 (hit) |  | 73.5 | 0.4 |
| CBK026431 |  | 13.7 | 0.4 |
| CBK028798 |  | 66.6 | 0.3 |
| CBK026288 |  | 3.2 | 0.1 |
| CBK027485 |  | 1.2 | 0.1 |
| CBK021573 |  | 1.4 | 0.1 |
| CBK024573 |  | 1.3 | 0.1 |
| CBK017849 |  | 6.6 | 0.1 |
| CBK017862 |  | 85.5 | 0.6 |
| CBK017858 |  | 2.0 | 0.1 |
| CBK017853 |  | 92.9 | 0.5 |
| CBK017852 |  | 93.0 | 0.5 |
| CBK017855 |  | 77.3 | 0.2 |
| CBK017848 |  | 1.2 | 0.1 |
| CBK018175 |  | 1.5 | 0.1 |
| CBK018098 |  | 58.7 | 0.2 |
| CBK017087 |  | 1.6 | 0.1 |
| CBK017621 |  | 7.6 | 0.1 |
| CBK010226 |  | 1.6 | 0.1 |
| CBK011516 |  | 84.7 | 0.7 |
| CBK009558 |  | 90.3 | 0.6 |
| CBK006380 |  | 1.3 | 0.1 |
| CBK006377 |  | 81 | 0.7 |
| CBK009814 |  | 6.2 | 0.1 |
| CBK092349 |  | 1.1 | 0.1 |
| CBK092364 |  | 2.3 | 0.1 |
| CBK092346 |  | 16 | 0.1 |
| CBK092341 |  | 1.8 | 0.1 |
| CBK092331 |  | 2.0 | 0.1 |
| CBK078118 |  | 3.2 | 0.1 |
| CBK085907 |  | 7.6 | 0.1 |
| CBK058133 |  | 84.5 | 0.4 |
| CBK057740 |  | 13.0 | 0.1 |
| CBK056955 |  | 10.8 | 0.1 |
| CBK027003 |  | 2.1 | 0.1 |
| CBK026341 |  | 15.2 | 0.1 |
| CBK026414 |  | 42.0 | 0.3 |
| CBK026412 |  | 53.4 | 0.3 |
| CBK026516 |  | 68.4 | 0.2 |
| CBK026406 |  | 25.9 | 0.2 |
| CBK017850 |  | 2.2 | 0.1 |
| CBK007143 |  | 1.3 | 0.1 |
| DMSO* |  | 1.5 | 0.0 |
| Epoxomicin** |  | 91.6 | 1.0 |

**Supplementary Table S2.1** **Secondary screen with structurally related compounds to the hit compound CBK092352** (as shown in Suppl. Fig. S2A). * = negative control. ** = positive control. The percentage (%) of positive cells was defined as cells with YFP levels in the nucleus above a predefined threshold (based on background fluorescence in DMSO-treated cells). The average YFP intensity in the nuclei of those cells classified as positive for YFP accumulation are shown as relative values to the positive control (epoxomicin-treated cells), set as 1.

| **Compound** | **Chemical structure** | **Percentage of YFP-positive cells (%)** | **Normalized average nuclear YFP intensity (positive cells)** |
| --- | --- | --- | --- |
| CBK260866 |  | 0.9 | 0.1 |
| CBK260867 |  | 0.5 | 0.1 |
| CBK260868 |  | 0.5 | 0.1 |
| CBK260869 |  | 0.3 | 0.1 |
| CBK260870 |  | 73.3 | 0.7 |
| CBK277750 |  | 85.7 | 0.4 |
| CBK277751 |  | 83.4 | 0.6 |
| CBK277752 |  | 0.2 | 0.0 |
| CBK277754 |  | 20.0 | 0.3 |
| CBK277755 |  | 2.3 | 0.2 |
| CBK277756 |  | 73.2 | 0.3 |
| CBK277757 |  | 75.9 | 0.3 |
| DMSO* |  | 0.0 | 0.0 |
| Epoxomicin** |  | 94.3 | 1.0 |

**Supplementary Table S2.2**. **Structure-activity relationship studies**. * = negative control. ** = positive control. The percentage (%) of positive cells was defined as cells with YFP levels in the nucleus above a predefined threshold (based on background fluorescence in DMSO-treated cells). The average YFP intensity in the nuclei of those cells classified as positive for YFP accumulation are shown as relative values to the positive control (epoxomicin-treated cells), set as 1.

| **Compound** | **Chemical structure** | **Percentage of YFP-positive cells (%)** | **Normalized average nuclear YFP intensity (positive cells)** |
| --- | --- | --- | --- |
| CBK277772 |  | 0.3 | 0.0 |
| CBK277775 |  | 1.7 | 0.0 |
| CBK277776 |  | 0.0 | 0.0 |
| CBK277778 |  | 91.5 | 0.4 |
| CBK277779 |  | 82.5 | 0.3 |
| CBK277781 |  | 89.2 | 0.3 |
| CBK277782 |  | 22.2 | 0.1 |
| DMSO* |  | 0.0 | 0.0 |
| Epoxomicin** |  | 98.16 | 1.0 |

**Supplementary Table S2.3**. **Structure-activity relationship studies**. * = negative control. ** = positive control. The percentage (%) of positive cells was defined as cells with YFP levels in the nucleus above a predefined threshold (based on background fluorescence in DMSO-treated cells). The average YFP intensity in the nuclei of those cells classified as positive for YFP accumulation are shown as relative values to the positive control (epoxomicin-treated cells), set as 1.

**Supplementary Table S3**. Excelsheet containing the results of the two replicates performed in the CRISPR/Cas9 screen, summarized per gene.

**Supplementary Table S4.**

| **Term ID** | **term description** | **Observed gene count** | **Background gene count** | **False discovery rate** | **Matching proteins in your network (labels)** |
| --- | --- | --- | --- | --- | --- |
| GO:0015037 | peptide disulfide oxidoreductase activity | 4 | 13 | 6.10e-06 | P4HB, PDIA3, PDIA4, TXN |
| GO:0003756 | protein disulfide isomerase activity | 3 | 20 | 0.00065 | P4HB, PDIA3, PDIA4 |
| GO: 0016209 | antioxidant activity | 4 | 79 | 0.00072 | GSTP1,  PRDX1,  PRDX4,  TXN |
| GO: 0004601 | peroxidase activity | 3 | 40 | 0.0025 | GSTP1, PRDX1, PRDX4 |
| GO: 0008379 | thioredoxin peroxidase activity | 2 | 5 | 0.0025 | PRDX1, PRDX4 |
| GO: 0043022 | ribosome binding | 3 | 50 | 0.0030 | EEF2, EIF6, RPSA |
| GO:0016853 | isomerase activity | 4 | 147 | 0.0034 | P4HB, PDIA3, PDIA4, PPIA |
| GO: 0008092 | cytoskeletal binding | 8 | 882 | 0.0036 | ANXA2, CNN3, DSTN, EEF2, HSP90AA1, MAP4, MARCKS, TPM3 |
| GO: 0051015 | actin filament binding | 4 | 158 | 0.0039 | DSTN, EEF2, MARCKS, TPM3 |
| GO: 0016491 | oxidoreductase activity | 7 | 716 | 0.0054 | GSTP1, P4HB, PDIA3, PDIA4, PRDX1, PRDX4, TXN |
| GO: 0003746 | translation elongation factor activity | 2 | 16 | 0.0074 | EEF1G, EEF2 |
| GO:0008135 | translation factor activity, RNA binding | 3 | 84 | 0.0079 | EEF1G, EEF2, EIF6 |
| GO:0003779 | actin binding | 5 | 413 | 0.0124 | CNN3, DSTN, EEF2, MARCKS, TPM3 |
| GO:000436 | glutathione transferase activity | 2 | 25 | 0.0130 | EEF1G, GSTP1 |
| GO:0005198 | structural molecule activity | 6 | 679 | 0.0160 | FLG2, MAP4, MYL6, RPSA, TPM3, TUBA1B |
| GO:0044877 | protein-containing complex binding | 7 | 968 | 0.0184 | DSTN, EEF2, EIF6, MARCKS, P4HB, RPSA, TPM3 |
| GO:0008307 | structural constituent of muscle | 2 | 45 | 0.0349 | MYL6, TPM3 |
| GO:0019205 | nucleobase-containing compound kinase activity | 2 | 49 | 0.0349 | NME2, TK1 |
| GO:0042802 | identical protein binding | 9 | 1754 | 0.0364 | ANXA2, HSP90AA1, LGALS1, MARCKS, PDIA3, PRDX1, PRDX4, S100A10, TK1 |
| GO:0050840 | extracellular matrix binding | 2 | 51 | 0.0364 | ANXA2, LGALS1 |
| GO:0019901 | protein kinase binding | 5 | 599 | 0.0394 | EEF2, GSTP1, HSP90AA1, MARCKS, PEBP1 |
| GO:0019899 | enzyme binding | 10 | 2197 | 0.0457 | ANXA2, ATOX1, EEF2, GSTP1, HSP90AA1, MARCKS, P4HB, PEBP1, TUBA1B, UBC |
| GO:0005488 | binding | 32 | 11878 | 0.0079 | ANXA2, ATOX1, CNN3, DSTN, DUT, EEF1G, EEF2, EIF6, FLG2, GSTP1, HCCS, HSP90AA1, LGALS1, MAP4, MARCKS, MYL6, NME2, P4HB, PCBP1, PDIA3, PEBP1, PRDX1, PRDX4, RPSA, S100A10, STRAP, TK1, TPM3, TUBA1B, UBC, VDAC2 |
| GO:0005515 | protein binding | 22 | 6605 | 0.0130 | ANXA2, ATOX1, CNN3, DSTN, EEF2, GSTP1, HSP90AA1, LGALS1, MAP4, MARCKS, P4HB, PDIA3, PEBP1, PPIA, PRDX1, PRDX4, S100A10, STRAP, TK1, TPM3, TUBA1B, UBC |

**Supplementary Table S4.** Enriched molecular function terms in the network of proteins enriched upon CBK77 treatment in the proteomics dataset.

**Supplementary Table S5**

| **Gene name** | **Gene ID** | **Catalog number** | **Company** | **Final concentration** | **Used in figure** |
| --- | --- | --- | --- | --- | --- |
| *UBC* | 7316 | L-019408-00-0005 | Dharmacon | 30 nM | Suppl. Fig. 4e |
| *VCP/p97* | 7415 | Custom: AACAGCCAUUCUCAAACAGAAUU | Dharmacon | 10 nM | Suppl. Fig. 4d |
| Silencer Select Negative Control #2 siRNA | NA | S814 | ThermoFisher Scientific (Invitrogen) | 30 nM | Suppl. Fig. 4e |
| AllStars negative control siRNA | NA | 1027281 | Qiagen | 10 nM | Fig. 5d |
| ON-TARGETplus non-targeting control | NA | D-001810-10 | Dharmacon | 10 nM | Suppl. Fig. 4d |
| *NQO1* | 1728 | L-005133-00 | Dharmacon | 10 nM | Suppl. Fig. 4d |
| *NQO1* | 1728 | J-005133-06 | Dharmacon | 10 nM | Fig. 5d, siNQO1#1 |
| *NQO1* | 1728 | J-005133-07 | Dharmacon | 10 nM | Fig. 5d, siNQO1#2 |
| *NQO1* | 1728 | J-005133-08 | Dharmacon | 10 nM | Fig. 5d, siNQO1#3 |
| *NQO1* | 1728 | J-005133-09 | Dharmacon | 10 nM | Fig. 5d, siNQO1#4 |
| *FAM129* | 116496 | L-013928-01 | Dharmacon | 10 nM | Suppl. Fig. 4d |
| *ANXA2* | 302 | L-010741-00 | Dharmacon | 10 nM | Suppl. Fig. 4d |
| *TUBA1B* | 10376 | L-020062-00 | Dharmacon | 10 nM | Suppl. Fig. 4d |
| *CFL1* | 1072 | L-012707-00 | Dharmacon | 10 nM | Suppl. Fig. 4d |
| *PCBP1* | 5093 | L-012243-01 | Dharmacon | 10 nM | Suppl. Fig. 4d |
| *MARCKS* | 4082 | L-004772-00 | Dharmacon | 10 nM | Suppl. Fig. 4d |
| *PRDX1* | 5052 | L-010338-00 | Dharmacon | 10 nM | Suppl. Fig. 4d |
| *P4HB* | 5034 | L-003690-00 | Dharmacon | 10 nM | Suppl. Fig. 4d |
| *LGALS1* | 3956 | L-011718-00 | Dharmacon | 10 nM | Suppl. Fig. 4d |
| *VCP* | 7415 | L-008727-00 | Dharmacon | 10 nM | Suppl. Fig. 4d |
| *DUT* | 1854 | L-010258-00 | Dharmacon | 10 nM | Suppl. Fig. 4d |
| *TXN* | 7295 | L-006340-00 | Dharmacon | 10 nM | Suppl. Fig. 4d |
| *TPM3* | 7170 | L-012784-00 | Dharmacon | 10 nM | Suppl. Fig. 4d |
| *PDIA3* | 2923 | L-003674-00 | Dharmacon | 10 nM | Suppl. Fig. 4d |
| *VDAC2* | 7417 | L-019766-00 | Dharmacon | 10 nM | Suppl. Fig. 4d |
| *PRDX4* | 10549 | L-012204-00 | Dharmacon | 10 nM | Suppl. Fig. 4d |
| *TUBB* | 203068 | L-010325-00 | Dharmacon | 10 nM | Suppl. Fig. 4d |
| *CNN3* | 1266 | L-011612-02 | Dharmacon | 10 nM | Suppl. Fig. 4d |
| *NME2* | 4831 | L-005102-00 | Dharmacon | 10 nM | Suppl. Fig. 4d |
| *S100A10* | 6281 | L-011766-00 | Dharmacon | 10 nM | Suppl. Fig. 4d |
| *STRAP* | 11171 | L-016282-01 | Dharmacon | 10 nM | Suppl. Fig. 4d |
| *MTPN* | 136319 | L-016817-01 | Dharmacon | 10 nM | Suppl. Fig. 4d |
| *DSTN* | 11034 | L-012303-00 | Dharmacon | 10 nM | Suppl. Fig. 4d |
| *CNN2* | 1265 | L-019793-02 | Dharmacon | 10 nM | Suppl. Fig. 4d |
| *YWHAZ* | 7534 | L-003332-00 | Dharmacon | 10 nM | Suppl. Fig. 4d |
| *EIF6* | 3692 | L-010096-00 | Dharmacon | 10 nM | Suppl. Fig. 4d |
| *PKM* | 5315 | L-006781-00 | Dharmacon | 10 nM | Suppl. Fig. 4d |
| *HSP90AA1* | 3320 | L-005186-00 | Dharmacon | 10 nM | Suppl. Fig. 4d |
| *TK1* | 7083 | L-006787-00 | Dharmacon | 10 nM | Suppl. Fig. 4d |
| *RPSA* | 3921 | L-013303-00 | Dharmacon | 10 nM | Suppl. Fig. 4d |
| *CPPED1* | 55313 | L-021262-02 | Dharmacon | 10 nM | Suppl. Fig. 4d |
| *PEBP1* | 5037 | L-019679-00 | Dharmacon | 10 nM | Suppl. Fig. 4d |
| *SOD1* | 6647 | L-008364-00 | Dharmacon | 10 nM | Suppl. Fig. 4d |
| *UBE2S* | 27338 | L-009707-00 | Dharmacon | 10 nM | Suppl. Fig. 4d |
| *HSPB1* | 3315 | L-005269-00 | Dharmacon | 10 nM | Suppl. Fig. 4d |
| *HSPA8* | 3312 | L-017609-00 | Dharmacon | 10 nM | Suppl. Fig. 4d |
| *ATOX1* | 475 | L-019059-01 | Dharmacon | 10 nM | Suppl. Fig. 4d |
| *EEF2* | 1938 | L-007245-00 | Dharmacon | 10 nM | Suppl. Fig. 4d |
| *GSTP1* | 2950 | L-011179-00 | Dharmacon | 10 nM | Suppl. Fig. 4d |
| *TUBA4A* | 7277 | L-008779-00 | Dharmacon | 10 nM | Suppl. Fig. 4d |

**Supplementary Table S5. siRNA oligonucleotides used in this study.** NA = non applicable.
